# Prognostic Value and Related Regulatory Networks of MRPL15 in Non-Small-Cell Lung Cancer

**DOI:** 10.3389/fonc.2021.656172

**Published:** 2021-05-07

**Authors:** Yangyang Zeng, Yingying Shi, Lu Xu, Yulan Zeng, Xiao Cui, Yuan Wang, Ningning Yang, Fuxiang Zhou, Yunfeng Zhou

**Affiliations:** ^1^ Hubei Key Laboratory of Tumor Biological Behaviors, Zhongnan Hospital of Wuhan University, Wuhan, China; ^2^ Department of Radiation and Medical Oncology, Zhongnan Hospital of Wuhan University, Wuhan, China; ^3^ Cancer Center, Union Hospital, Tongji Medical College, Huazhong University of Science and Technology, Wuhan, China

**Keywords:** MRPL15, non-small-cell lung cancer, prognostic value, functional network analysis, immune infiltration

## Abstract

**Background:**

Mitochondrial ribosomal protein L15 (MRPL15), a member of mitochondrial ribosomal proteins whose abnormal expression is related to tumorigenesis. However, the prognostic value and regulatory mechanisms of MRPL15 in non-small-cell lung cancer (NSCLC) remain unclear.

**Methods:**

GEPIA, ONCOMINE, Gene Expression Omnibus (GEO), UALCAN, Kaplan–Meier plotter, PrognoScan, LinkedOmics and GeneMANIA database were utilized to explore the expression and prognostic value of MRPL15 in NSCLC. Additionally, immune infiltration patterns were evaluated *via* ESTIMATE algorithm and TISIDB database. Furthermore, the expression and prognostic value of MRPL15 in lung cancer were validated *via* immunohistochemistry (IHC) assays.

**Results:**

In NSCLC, multiple cohorts including GEPIA, ONCOMINE and 8 GEO series (GSE8569, GSE101929, GSE33532, GSE27262, GSE21933, GSE19804, GSE19188, GSE18842) described that MRPL15 was up-regulated. Moreover, MRPL15 was notably linked to gender, clinical stage, lymph node status and the TP53 mutation status. And patients with high MRPL15 expression showed poor overall survival (OS), progression-free survival (PFS), disease-free survival (DFS) and relapse-free survival (RFS) in NSCLC. Then, functional network analysis suggested that MRPL15 participated in metabolism-related pathways, DNA replication and cell cycle signaling *via* pathways involving several kinases, miRNAs and transcription factors. Additionally, it was found that MRPL15 expression was negatively related to immune infiltration, including immune scores, stromal scores and several tumor-infiltrating lymphocytes (TILs). Furthermore, IHC results further confirmed the high MRPL15 expression and its prognostic potential in lung cancer.

**Conclusions:**

These findings demonstrate that high MRPL15 expression indicates poor prognosis in NSCLC and reveal potential regulatory networks as well as the negative relationship with immune infiltration. Thus, MRPL15 may be an attractive predictor and therapeutic strategy for NSCLC.

## Introduction

Lung cancer has been regarded as the leading cause of cancer deaths worldwide (1), in which NSCLC with aggressive clinical course and prominent association with tobacco use (2) is the main histological type (3). NSCLC includes several subtypes, among which lung adenocarcinoma (LUAD) and lung squamous cell carcinoma (LUSC) are the most prevalent. Due to a high proportion of patients with NSCLC are already metastatic at diagnosis, novel biomarkers that can reflect clinical status are urgently needed.

Alterations of mitochondrial DNA (mtDNA) possibly are related to carcinogenesis of lung cancer (4, 5). More and more data have revealed that tumorigenesis is partially dependent on the reprogramming of cellular metabolism as consequence of mitochondrial dysfunction (6, 7). MtDNA mutation may contribute to malignant transformation *via* ROS and mitochondrial metabolites (8, 9). Mitochondria not only can promote progression, but also functions in cell death signaling (10). Thus, mitochondria are supposed to be the regulators of cellular life and death in tumor cells. 13 proteins encoded by mtDNA (11), all of which are the mitochondrial respiratory chain enzymes. These proteins are synthesized in specialized mitochondrial ribosomes (mitoribosomes), which are composed of two rRNAs and mitochondrial ribosomal proteins (MRPs) (12).

Mutations in nuclear-encoded MRPs can lead to severe respiratory chain dysfunction (13) and multiple MRPs are important predictors of tumor diagnosis. Almost 80 MRP genes have been recognized, of which are divided into two main groups: components of the large subunit (MRPL) and components of the small subunit (MRPS) (14). Multiple MRPs abnormalities can be found in the same cancer. For instance, both MRPS30 and MRPL13 had been reported to be overexpressed and correlate to poor survival in breast cancer (15). Meanwhile, the same MRPs might work in various cancers. High MRPL13 promoted invasion in liver cancer cells (16). Apart from participating in mitochondrial oxidative phosphorylation (OXPHOS), the new roles for MRPs in cellular apoptosis and proliferation have been revealed (17). Actually, research has declared that MRPS29 promotes apoptosis through its interactions with Fas ligand (18).

MRPL15 is a member of MRPL, which provides energy in the form of ATP for cell growth. MRPL15 has been reported to have a predictive value in breast cancer metastasis (19) and can promote Burkitt lymphoma growth (20). Little research has reported the predictive value of MRPL15 in NSCLC. Only a recent study showed that MRPL15 was associated with progression in lung adenocarcinoma (21), but the prognostic value and regulatory mechanisms of MRPL15 in lung cancer are still unknown. In addition, immunological analysis of the tumor microenvironment shows great promise for better prognosis and immunotherapy benefit for NSCLC (22). However, few studies reveal the relationship between MRPs and immune infiltration patterns.

## Methods

### GEPIA

GEPIA (http://gepia.cancer-pku.cn/) is a web-based tool providing key interactive and customizable functions based on The Cancer Genome Atlas (TCGA) and the Genotype-Tissue Expression (GTEx) data (23). In this study, we explored the differential mRNA expression levels of MRPL15 in tumor and normal tissue datasets, especially LUAD and LUSC.

### ONCOMINE

The mRNA expression of MRPL15 was assessed in lung carcinoma in the ONCOMINE database (www.oncomine.org), a cancer microarray database and integrated data-mining platform (24). This analysis drew on a study using the Hou Lung dataset (25).

### Data Collection

Eight expression microarray series GSE8569, GSE101929, GSE33532, GSE27262, GSE21933, GSE19804, GSE19188 and GSE18842 containing NSCLC tumor and normal tissues with MRPL15 expression information were collected from the Gene Expression Omnibus dataset (GEO, https://www.ncbi.nlm.nih.gov/geo/). Information about selected GEO series is listed in **Table 1**. All the download datasets included NSCLC tumor and normal tissues, and the total number of samples in each dataset was more than 30.

**Table 1:** Information of GEO series included in this analysis.

GEO series	Contributor(s)	Tumor	Normal	Platform
GSE8569	Angulo et al., 2007	69	6	CNIO Human Oncochip 2.0
GSE101929	Mitchell et al., 2017	32	34	Affymetrix Human Genome U133 Plus 2.0 Array
GSE33532	Meister et al., 2011	80	20	Affymetrix Human Genome U133 Plus 2.0 Array
GSE27262	Li-Jen et al., 2011	25	25	Affymetrix Human Genome U133 Plus 2.0 Array
GSE21933	Chang et al., 2010	21	21	Phalanx Human OneArray
GSE19804	Lu et al., 2010	60	60	Affymetrix Human Genome U133 Plus 2.0 Array
GSE19188	Philipsen, 2009	91	65	Affymetrix Human Genome U133 Plus 2.0 Array
GSE18842	Ramos et al., 2019	46	45	Affymetrix Human Genome U133 Plus 2.0 Array

### UALCAN Database Analysis

UALCAN (http://ualcan.path.uab.edu) is a web portal to perform tumor subgroup gene expression and survival analyses based on TCGA data (26). Therefore, we analyzed the relative expression of MRPL15 in various tumor sub-groups based on gender, clinical stage, lymph node status and the TP53 mutation status of LUAD and LUSC.

### Survival Analysis

The Kaplan–Meier plotter (http://kmplot.com/analysis/) (27) was applied for survival analysis of MRPL15 in lung cancer patients according to the hazard ratio (HR) with 95% confidence interval (CI) and log-rank P-values. GEPIA was also used for survival analysis. In addition, the prognostic potential of MRPL15 was validated in the PrognoScan database (http://www.abren.net/PrognoScan/) (28) with Cox P-value <0.05 as the threshold.

### LinkedOmics Analysis

LinkedOmics (http://www.linkedomics.org/login.php) is a platform for analyzing cancer multi-omics data based on TCGA project (29). The LinkFinder module was applied to investigate the relationship of MRPL15 and differentially expressed genes in the LUAD cohort (n = 389, selected patients) and LUSC cohort (n = 319, selected patients) from TCGA, respectively. Moreover, the LinkInterpreter module was used to reveal potential regulatory mechanisms through pathway and network analysis. Gene set enrichment analysis (GSEA) tool was used to conduct KEGG pathways, kinase-target enrichment, miRNA-target enrichment and transcription factor-target enrichment. The rank standard was FDR <0.05 and 500 simulations.

### GeneMANIA Analysis

GeneMANIA (http://www.genemania.org) is a website for generating hypotheses about gene function, analyzing gene lists and prioritizing genes for functional assays (30), such as information for protein and genetic interactions, enrichment analysis and so on. GeneMANIA was used to construct gene-gene functional interaction networks among the genes that GSEA identified as being enriched in LUAD and LUSC: kinase HCK and transcription factor ELK1.

### Tumor Immunology Analysis

The immune scores and stromal scores of each case were analyzed by Estimation of STromal and Immune cells in MAlignant Tumor tissues using Expression data (ESTIMATE) algorithm. Further, the different gene expression levels of human leukocyte antigen (HLA) family genes between the high-expression group and the low-expression group of MRPL15 were analyzed based on TCGA.

TISIDB (http://cis.hku.hk/TISIDB) is a web portal for investigation of tumor-immune interactions, which contains multiple types of data resources in oncoimmunology (31). TISIDB was employed to investigate correlations between MRPL15 expression and lymphocytes.

### Tissue Microarray (TMA) and IHC

The tissue microarray containing 92 lung cancer tissues and 88 adjacent normal tissues was purchased from Shanghai Outdo Biotech (Shanghai, China). IHC staining was performed as previously described (32) with mouse monoclonal MRPL15 antibody (OriGene Technologies Inc., TA807480, 1:300 dilution). The staining results were identified by integrated scoring: the immunostaining intensity (negative, no staining of cells = 0; weak staining = 1; moderate staining = 2; strong staining = 3) and the percentage of the positively stained area (0–25% = 1, 26–50% = 2, 51–75% = 3, >75% = 4). The score was finally calculated according to the above staining criteria, and the scores over 6 were regarded as high expression.

### Statistical Analysis

Statistical analysis was carried out by SPSS 23.0 (IBM, Armonk, NY, USA) and GraphPad Prism 8.0 (La Jolla, CA, USA) software. The *t*-test was used for differential MRPL15 expression analysis and log-rank test was used to perform group comparison of survival correlation. Furthermore, the relationship between MRPL15 expression and clinicopathologic features was analyzed by Chi-square and independent prognostic factors of lung cancer patients were confirmed by the Cox regression analyses. The differences were considered significant when the probability value was less than 0.05.

## Results

### Up-Regulated Expression of MRPL15 in NSCLC

**Figure 1:**
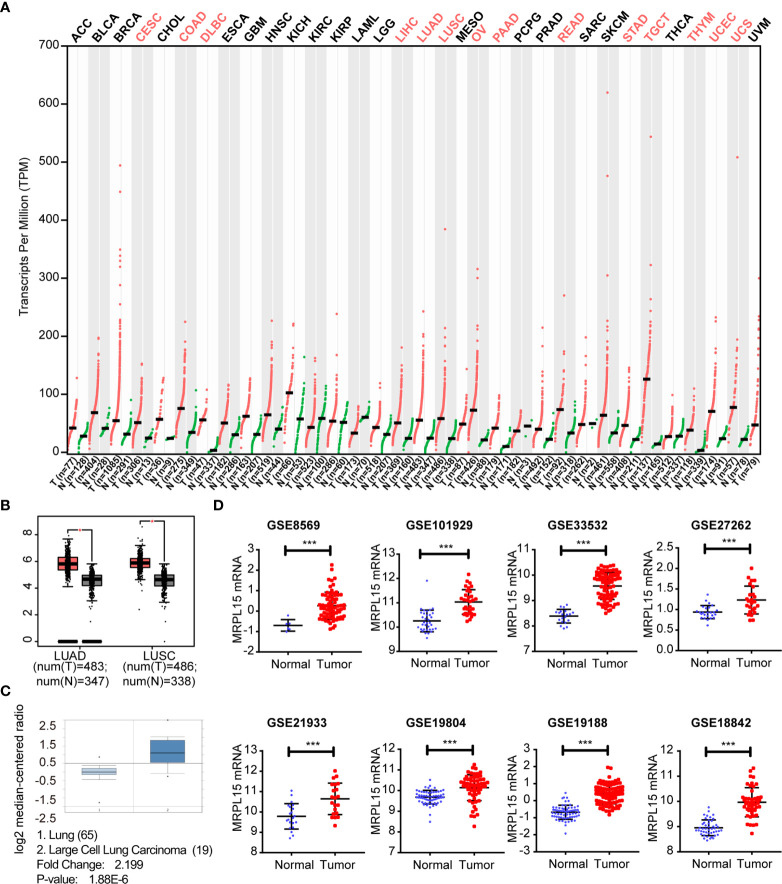
Up-regulated expression of MRPL15 in NSCLC. **(A)** The expression levels of MRPL15 in pan-cancer. Data was obtained from the GEPIA. **(B)** The expression of MRPL15 in LUAD and LUSC in GEPIA. **(C)** The mRNA levels of MRPL15 in the Hou Lung dataset. **(D)** MRPL15 expression levels between tumor tissues and adjacent normal tissues in patients with NSCLC in eight GEO series (***P < 0.001).

We initially examined the level of MRPL15 expression in multiple tumor tissues in GEPIA. The analysis revealed that MRPL15 was up-regulated in pan-cancer (**Figure 1A**). Then, we further evaluated MRPL15 expression in multiple NSCLC cohorts from GEPIA, Oncomine and GEO databases. The expression of MRPL15 in LUAD or LUSC was obviously higher than in non-tumor tissues (**Figure 1B**). Meanwhile, MRPL15 ranked within the top 5% up-regulated genes in large cell lung carcinoma based on mRNA expression in Oncomine database (**Figure 1C**). That MRPL15 was also significantly overexpressed in GSE8569, GSE101929, GSE33532, GSE27262, GSE21933, GSE19804, GSE19188 and GSE18842 datasets (**Figure 1D**). The information of eight GEO series was shown in **Table 1**. The results demonstrated that MRPL15 was significantly elevated in NSCLC tissues.

### The Prognostic Value of MRPL15 and Related Clinic-Pathological Characters

**Figure 2:**
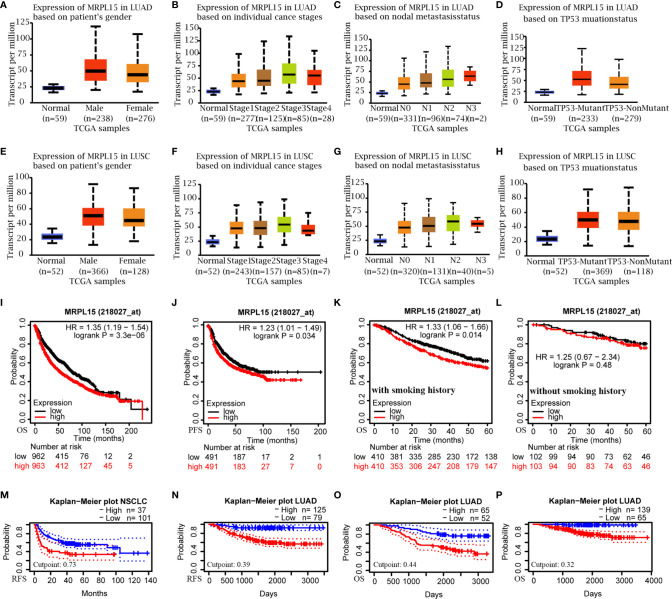
The sub-group analysis of multiple clinic-pathological features (UALCAN) and the prognostic value of MRPL15 in NSCLC. **(A–D)** Relative expression of MRPL15 in healthy and LUAD people with different **(A)** gender, **(B)** clinical stage, **(C)** lymph node status and **(D)** TP53 mutation status; **(E–H)** Relative expression of MRPL15 in healthy and LUSC people with different **(E)** gender, **(F)** clinical stage, **(G)** lymph node status and **(H)** TP53 mutation status. **(I, J)** Overall survival (OS) and progression-free survival (PFS) of MRPL15 in lung cancer cohort. **(K, L)** Subgroup analyses of 5-year OS comparison in **(K)** the group with smoking history and **(L)** the other group without smoking history of lung cancer patients. **(M, N)** Relapse-free survival (RFS) in the NSCLC cohort (GSE8894, n = 138, **M**) and LUAD cohort (GSE31210, n = 204**, N**). **(O, P)** OS in two LUAD cohorts [GSE13213 (n = 117, **O**) and GSE31210 (n = 204, **P**)].

Multiple clinic-pathological characters of LUAD and LUSC samples in the TCGA were further analyzed. The results of subgroup analyses claimed that MRPL15 was dramatically higher in LUAD or LUSC patients than in healthy people within different gender, clinical stage, lymph node status and the TP53 mutation status (**Figures 2A–H**). Furthermore, the up-regulated MRPL15 was notably correlated with poor OS (HR = 1.35, log rank P = 3.30E−06, **Figure 2I**), PFS (HR = 1.23, log rank P = 0.034, **Figure 2J**) and DFS (HR = 1.3, log rank P = 0.025, **Supplementary Figure 1**) in NSCLC patients. Interestingly, high MRPL15 expression was connected with worse 5-year OS in NSCLC patients with smoking history (**Figure 2K**), rather than in NSCLC without smoking history (**Figure 2L**). Additionally, data in NSCLC cohort (GSE8894, **Figure 2M**) and LUAD cohort (GSE31210, **Figure 2N**) ld> revealed that high MRPL15 expression was also associated with poor RFS, indicating an aggressive role for MRPL15. That high MRPL15 was positively linked with OS was also supported by two LUAD cohort (GSE13213, GSE31210, **Figures 2O, P**).

### Differentially Expressed Genes Associated With MRPL15 in NSCLC

**Figure 3:**
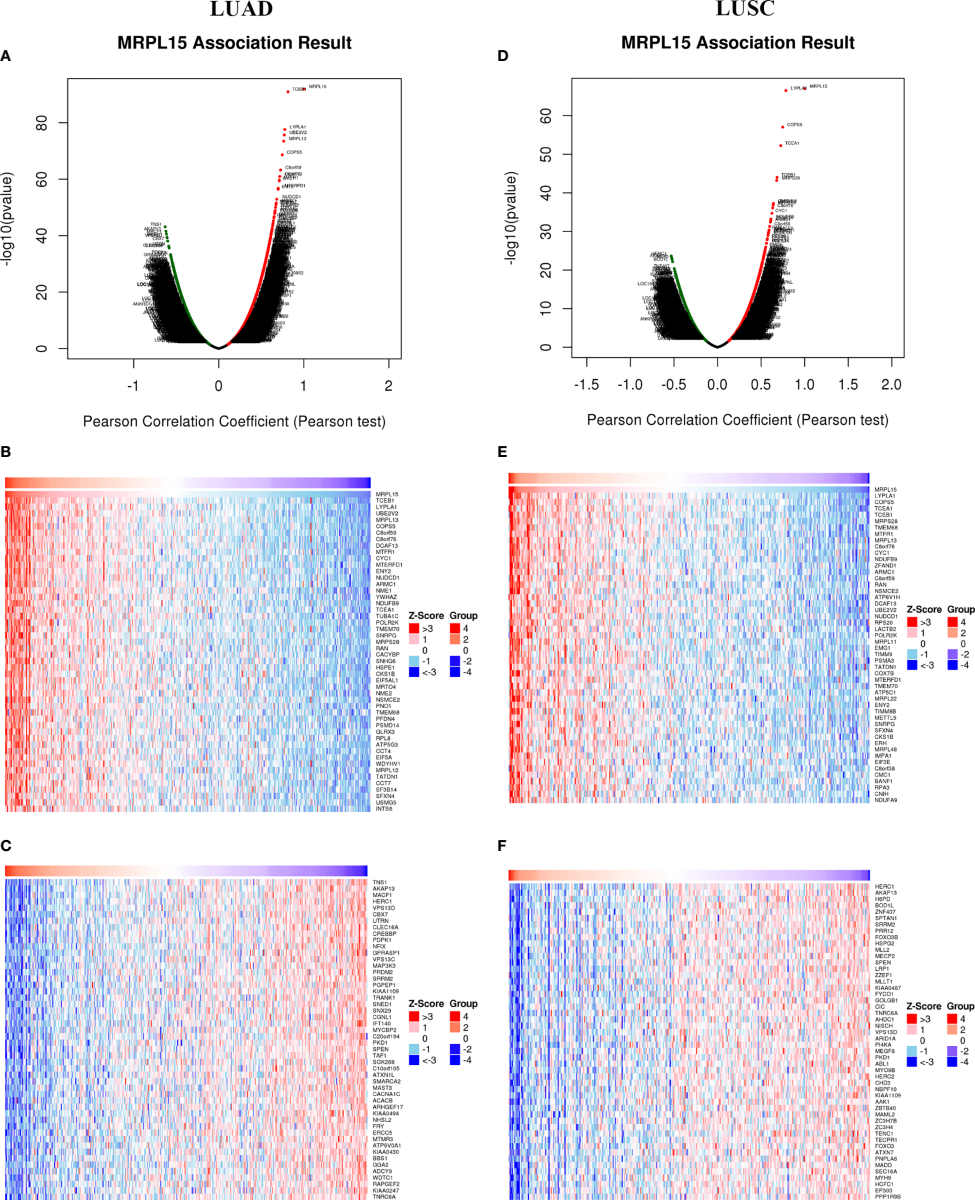
Differentially expressed genes associated with MRPL15 in NSCLC (LinkedOmics). **(A)** Using Pearson test to identify significantly expressed genes related to MRPL15 in LUAD. **(B, C)** TOP 50 remarkable genes associated with MRPL15 in LUAD. **(D)** Using Pearson test to identify significantly expressed genes related to MRPL15 in LUSC. **(E, F)** TOP 50 remarkable genes associated with MRPL15 in LUSC. Red represents positively correlated genes and green represents negatively correlated genes.

To explore the biological function of MRPL15 in NSCLC, the LinkedOmics was applied to investigate co-expression genes associated with MRPL15 in LUAD (N = 389 patients) and LUSC cohort (N = 319 patients). In LUAD (**Figure 3A**), 3,987 genes were positively correlated with MRPL15, whereas 6,681 genes were negatively correlated (false discovery rate, FDR <0.01). And in LUSC (**Figure 3D**), 2,859 genes were positively correlated with MRPL15, whereas 4,286 genes were negatively correlated. The top 50 remarkable genes related to MRPL15 in LAUD (**Figures 3B, C**) and LUAD (**Figures 3E, F**) were displayed in the heat map, suggesting an extensive influence of MRPL15 on global transcriptome. Lysophospholipase1 (LYPLA1) was the most positively significant gene connected with MRPL15 expression in NSCLC (rank #2 in LUAD, R = 0.776, P = 2.44E−78; rank #1 in LUSC, R = 0.785, P = 3.12E−67). LYPLA1 was known as acyl−protein thioesterase1 (APT1), and its suppression inhibited proliferation and invasion in NSCLC cells (33).

### KEGG Pathways Networks of MRPL15 in NSCLC

**Figure 4:**
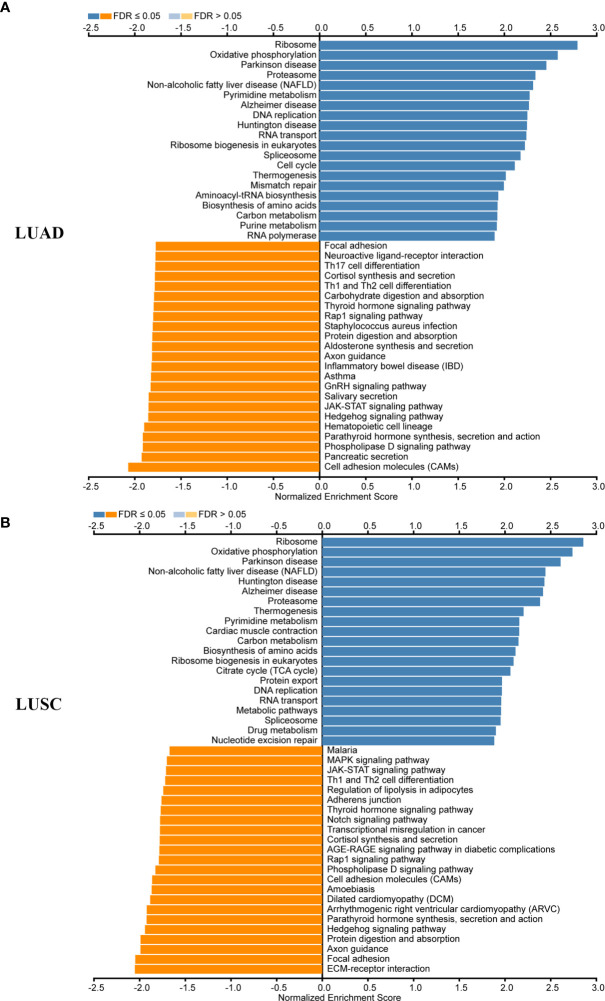
KEGG pathways networks of MRPL15 in NSCLC. **(A)** KEGG enrichments of MRPL15 in LUAD cohort. **(B)** KEGG enrichments of MRPL15 in LUSC cohort.

In LUAD and LUSC, KEGG pathway analysis showed that MRPL15 co-expressed genes participate in metabolism function (like oxidative phosphorylation, carbon metabolism and pyrimidine metabolism) and DNA replication (**Figures 4A, B**). Higher expression of pyrimidine synthesis genes was shown to result in poor prognosis of patients with glioblastoma (34) and NSCLC (35, 36). As MRPL15 is important for mitochondrial metabolism, we have reason to suppose that MRPL15 is likely associated with pyrimidine metabolism. Moreover, some immune-related activities like Th1 and Th2 cell differentiation and Th17 cell differentiation were inhibited (**Figures 4A, B**). The above results indicate that MRPL15 may play a positive role in metabolism function, while a negative role in immune infiltration in NSCLC.

### Regulators of MRPL15 Networks in NSCLC

Next, we explored the regulators enrichment of MRPL15 co-expressed genes in NSCLC, including kinases, miRNAs and transcription factors’ (TF) enrichment. In LUAD, the kinases-target network was associated with hematopoietic cell kinase (HCK), polo like kinase 1 (PLK1), Aurora kinase B (AURKB), polo like kinase 3 (PLK3), and cyclin-dependent kinase 1 (CDK1) (**Table 2** and **Supplementary Table 1**). In LUSC, the kinases-target network was mainly related to hematopoietic cell kinase (HCK) , mitogen-activated protein kinase 7 (MAPK7), protein kinase AMP-activated catalytic subunit alpha 2 (PRKAA2), mitogen-activated protein kinase 10 (MAPK10), mitogen-activated protein kinase 3 (MAPK3) (**Table 2** and **Supplementary Table 2**). The miRNAs enriched in LUAD and LUSC were shown in **Table 2** and **Supplementary Tables 3**, **4**, while transcription factor enriched in LUAD and LUSC were listed in **Table 2** and **Supplementary Tables 5**, **6**.

**Table 2:** The Kinase, miRNA and transcription factor-target networks of MRPL15 in NSCLC (LinkedOmics).

Enriched Category	LUAD				LUSC		
	Geneset	LeadingEdgeNum		FDR	Geneset	LeadingEdgeNum	FDR
**Kinase Target**	Kinase_HCK		14	0	Kinase_MAPK7	17	0.026220
	Kinase_PLK1		33	0.001544	Kinase_HCK	13	0.026220
	Kinase_AURKB		35	0.006952	Kinase_PRKAA2	11	0.030590
	Kinase_PLK3		10	0.011844	Kinase_MAPK10	9	0.030590
	Kinase_CDK1		85	0.018153	Kinase_MAPK3	67	0.031561
**miRNA Target**	ATGTACA,MIR-493		115	0	TTTGCAC,MIR-19A,MIR-19B	178	0
	GTGCAAA,MIR-507		51	0	GTGCAAA,MIR-507	49	0
	TTTGCAC,MIR-19A,MIR-19B		162	0	TTGCACT,MIR-130A,MIR-301,MIR-130B	144	0
	GCACCTT,MIR-18A,MIR-18B		53	0	TGCACTT,MIR-519C,MIR-519B,MIR-519A	149	0
	TGCACTT,MIR-519C,MIR-519B,MIR-519A		124	0	GCACCTT,MIR-18A,MIR-18B	52	0
	GCACTTT,MIR-17-5P,MIR-20A,MIR-106A,MIR-106B,MIR-20B,MIR-519D		185	0	GCACTTT,MIR-17-5P,MIR-20A,MIR-106A,MIR-106B,MIR-20B,MIR-519D	202	0
	GAGCCTG,MIR-484		45	0	GAGCCTG,MIR-484	60	0
	TTGCACT,MIR-130A,MIR-301,MIR-130B		124	0	ATGTACA,MIR-493	121	0
**Transcription Factor Target**	V$ELK1_02		72	0	GGAANCGGAANY_UNKNOWN	39	0
	SCGGAAGY_V$ELK1_02		348	0	SCGGAAGY_V$ELK1_02	316	2.82E-04
	GGAANCGGAANY_UNKNOWN		39	0	TMTCGCGANR_UNKNOWN	55	8.47E-04
	TMTCGCGANR_UNKNOWN		44	0	V$ELK1_02	75	9.42E-04
	V$DBP_Q6		68	0	AAAYWAACM_V$HFH4_01	77	0.001939

****LeadingEdgeNum, the number of leading edge genes; FDR, false discovery rate from Benjamini and Hochberg from gene set enrichment analysis (GSEA). V$, the annotation found in Molecular Signatures Database (MSigDB) for transcription factors (TF).

**Figure 5:**
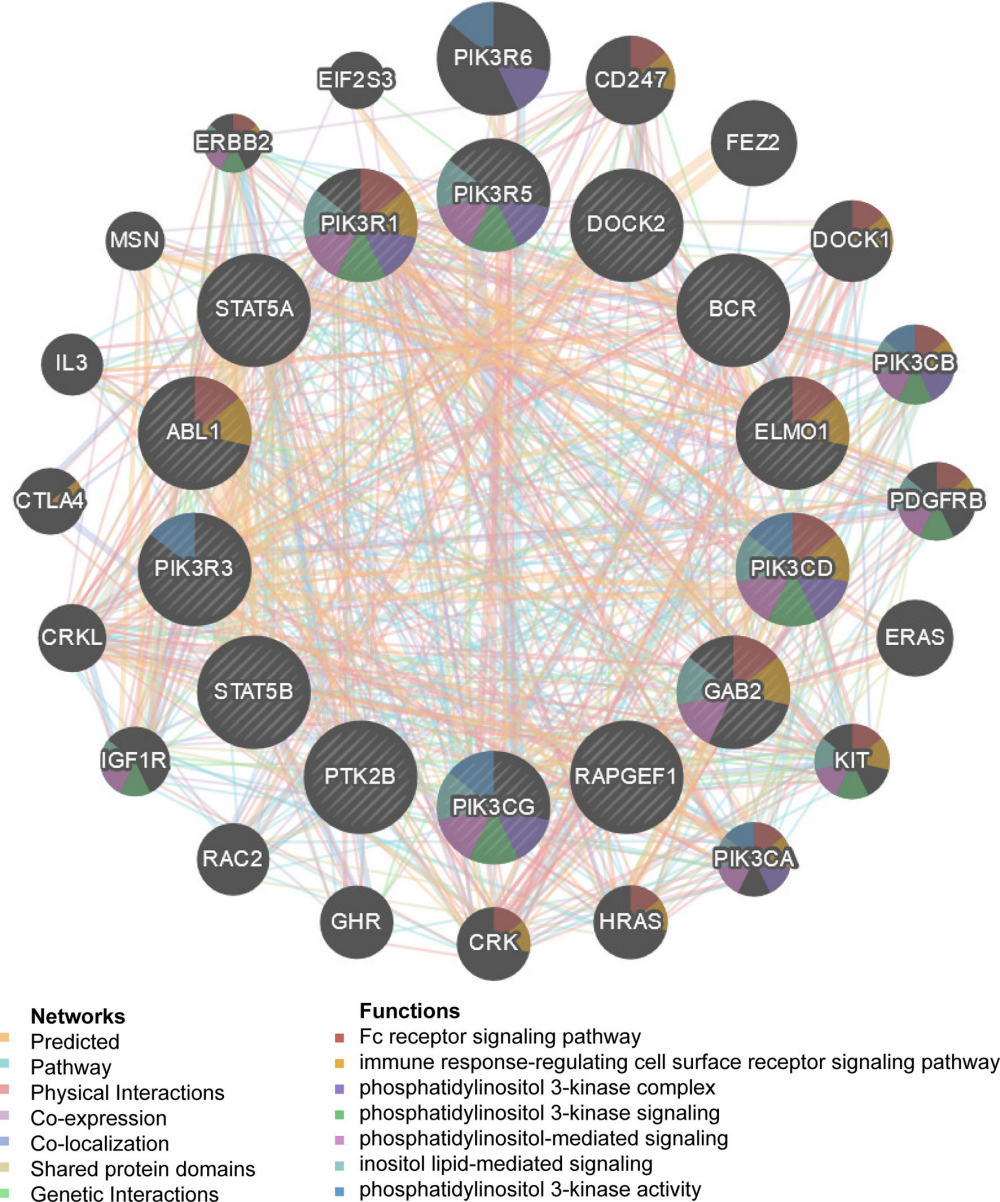
Protein–protein interaction network of kinases HCK-target networks in LUAD (GeneMANIA). PPI network and functional analysis indicating the gene set that was enriched in the target network of HCK. The network nodes’ colors represent the biological functions of the enrichment genes.

**Figure 6:**
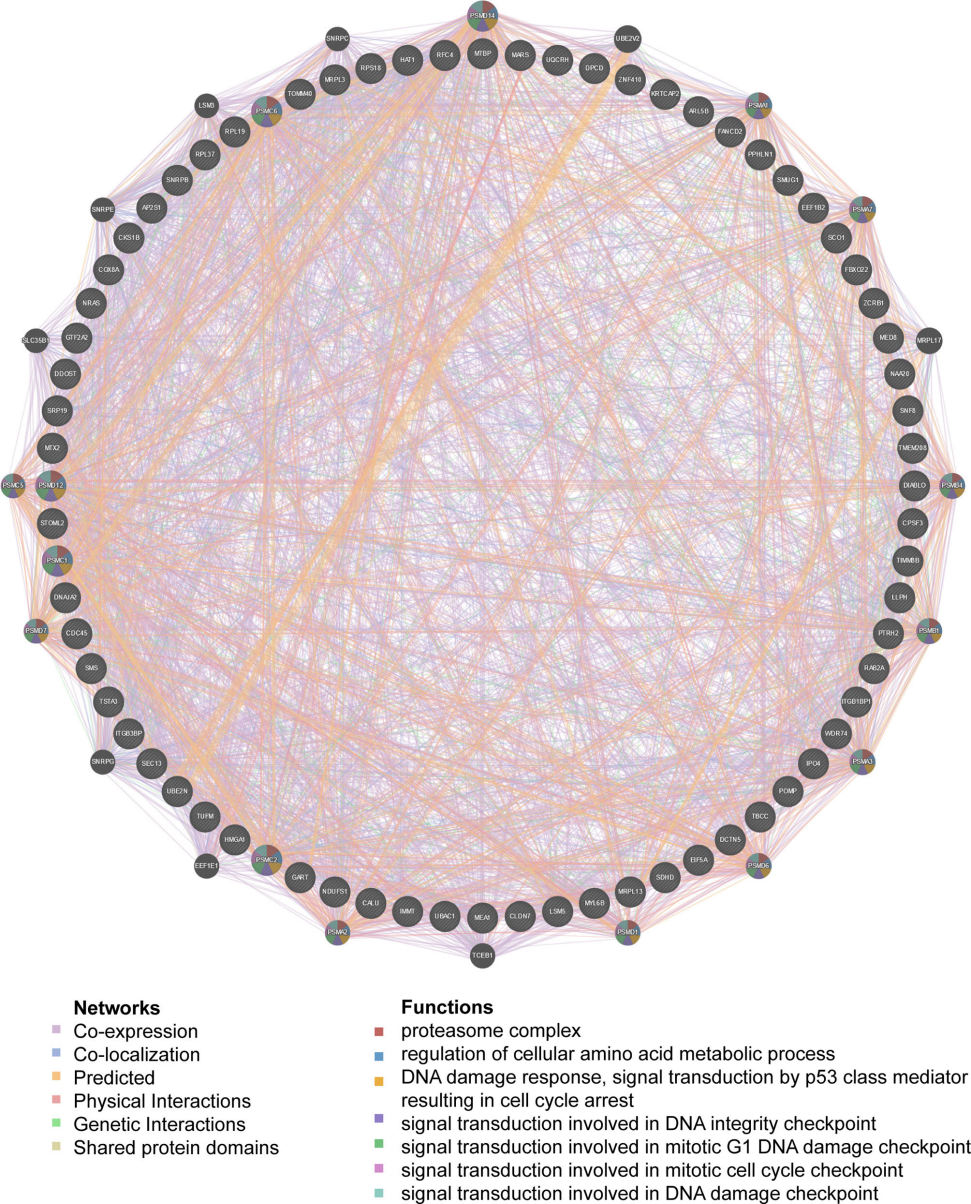
PPI network of transcription factor ELK1-target networks in LUAD (GeneMANIA). PPI network and functional analysis indicating the gene set that was enriched in the target network of ELK1-target. The network nodes’ colors represent the biological functions of the enrichment genes.

We observed that HCK and ETS-like 1 transcription factor (ELK1) were in the top five regulators enriched in both LUAD and LUSC. Using protein- protein interaction (PPI) network, we revealed that kinase HCK regulating Fc receptor signaling pathway, immune response-regulating cell surface receptor signaling pathway and phosphatidylinositol 3-kinase complex were enriched in LUAD (**Figure 5**) or LUSC (**Supplementary Figure 2**). Inhibition of HCK has been reported to suppress macrophage polarization and impairs gastric tumor growth (37). Meanwhile, HCK knockdown can enhance the antitumor effects of immunotoxin (38). Taken together, the results indicate that MRPL15 may affect the process of immune through HCK signaling pathway. Additionally, the gene set enriched for transcription factor ELK1 (TF V$ELK1_02) included regulation of cellular amino acid metabolism, DNA damage response, signal transduction by p53 class mediator resulting in cell cycle arrest and signal transduction involved in mitotic DNA integrity checkpoint in LUAD (**Figure 6**) or LUSC (**Supplementary Figure 3**). ELK1 could promote epithelial to mesenchymal transition (EMT) in osteosarcoma tumor cell (39) and NSCLC (40). Moreover, MER/ERK/ELK1 pathway has been shown to promote amino acid metabolism (41, 42) and inactivation of ELK1 results in elevated apoptosis in response to viral infection (43). These findings confirm that HCK and ELK1 may participate in the metabolism function involved in the process that MRPL15 promotes tumorigenesis in NSCLC.

### MRPL15 Expression Is Linked to Immune Infiltration Level

**Figure 7:**
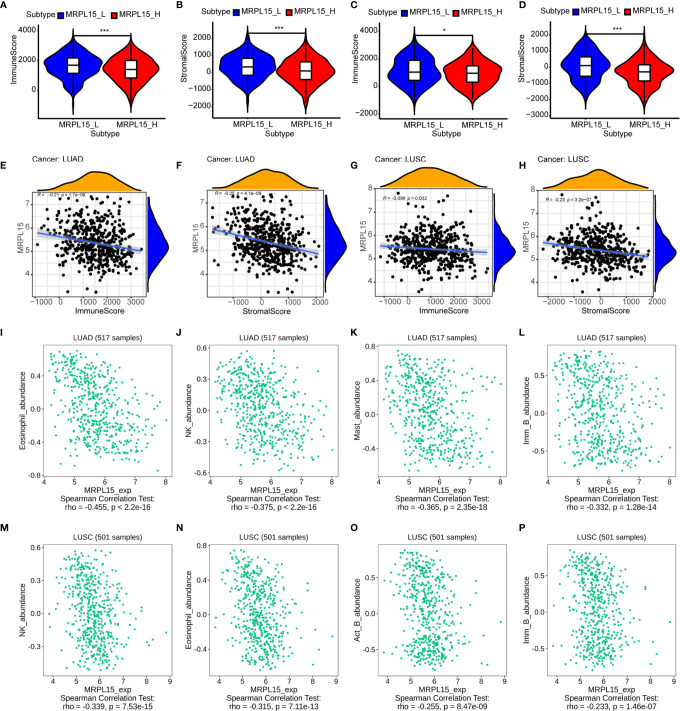
Association of MRPL15 expression with immune cell infiltration levels. **(A, B)** The high-expression group showed lower **(A)** immune score and **(B)** stromal score than the low-expression group in LUAD. **(C, D)** The high-expression group showed lower **(C)** immune score and **(D)** stromal score than the low-expression group in LUSC *P < 0.05, **P < 0.01, ***P < 0.001. **(E, G)** Association between immune score and the expression of MRPL15 in **(E)** LUAD and **(G)** LUSC. **(F, H)** Association between stromal score and the expression of MRPL15 in **(F)** LUAD and **(H)** LUSC. **(I–L)** Top four TILs displaying the greatest Spearman’s correlation with MRPL15 expression in LUAD. **(M–P)** Top four TILs displaying the greatest Spearman’s correlation with MRPL15 expression in LUSC.

In lung cancer, immunological parameters were reported to better predict the clinical outcome than TNM stage (44). To investigate whether MRPL15 expression was correlated with immune infiltration patterns in NSCLC, we compared the degree of immune cell infiltration between the high-expression group and the low-expression group by ESTIMATE algorithm. The immune score and stromal score were lower in the high-expression group than the low-expression group in LUAD **(** **Figures 7A, B**) and LUSC (**Figures 7C, D**). Besides, the expression of MRPL15 was significantly negatively correlated with the immune score and stromal score in LUAD (**Figures 7E, F**) and LUSC (**Figures 7G, H**). Our results showed the correlation of MRPL15 expression level with poorer prognosis and low immune infiltration in NSCLC. Due to human leukocyte antigen (HLA) holding a predictive role in developing immune-related adverse events (irAEs) during therapy in NSCLC (45), we also investigated significantly lower expression level of the human leukocyte antigen (HLA) family genes in high-expression MRPL15 group in LUAD and LUSD (**Supplementary Figures 4A, B**). Moreover, Spearman’s correlations between MRPL15 expression and lymphocytes were analyzed using the TISIDB database. The top four TILs negatively associated with MRPL15 expression were eosinophils, natural killer cells, mast cells and immature B cells in LUAD (**Figures 7I–L**), while eosinophils, natural killer cells, activated B cell and immature B cells in LUSC (**Figures 7M–P**). Our findings suggest that MRPL15 may play a significant role in immune infiltration in NSCLC.

### The Prognostic Value of MRPL15 Expression in Lung Cancer Patients

**Figure 8:**
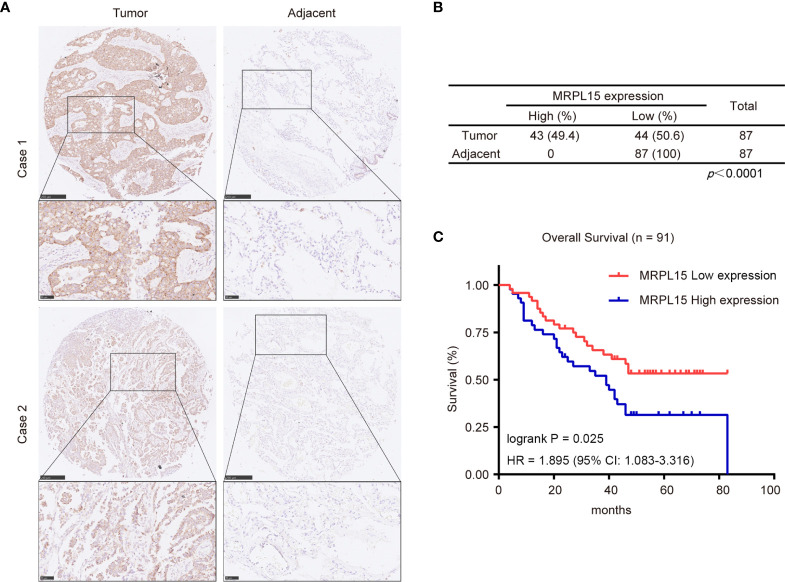
Expression levels of MRPL15 in microarrays. **(A)** Representative pictures of MRPL15 IHC in lung cancer tissues and matched adjacent normal tissues. **(B)** Statistical analysis of IHC staining in lung cancer tissues and matched adjacent normal tissues. **(C)** Kaplan–Meier curves displaying OS for MRPL15 protein expression in the samples included in the analysis.

In this study, 91 lung cancer patients with complete clinical information were included in the analysis and the clinical characteristics were shown in **Table 3**. Our results indicated that MRPL15 expression is strongly related to lymph node metastasis (P = 0.028). As shown in **Figures 8A, B**, the specific staining was observed mainly in the cytoplasm and the expression scores of MRPL15 were significantly higher in tumor tissues than in adjacent non-tumorous tissues. The lung cancer patients with high MRPL15 expression had a poor OS than those with low MRPL15 expression (**Figure 8C**). Furthermore, multivariate analysis revealed that MRPL15 expression level [hazard ratio (HR): 1.996; 95% CI: 1.121–3.554; P = 0.019] remained potential independent prognostic factors (**Table 4**). Taken together, MRPL15 can serve as an independent risk factor for survival and prognosis of lung cancer patients.

**Table 3:** Correlation between MRPL15 expression and clinicopathological characteristics in lung cancer patients.

Characteristics	total	MRPL15 expression		Chi-square	*P*
Patient	91	low	high		
		48	43		
**Age**					0.104
<60	33 (36.3%)	14 (29.2%)	19 (44.2%)	2.21	0.137
≥60	58 (63.7%)	34 (70.8%)	24 (55.8%)
**Gender**					
Male	51 (44%)	30 (37.5%)	21 (51.2%)	1.719	0.19
Female	40 (56%)	18 (62.5%)	22 (48.8%)
**Pathological type**					
Adenocarcinoma	67 (73.6%)	31 (64.6%)	36 (83.7%)	4.33	0.115
Bronchioloalveolar Carcinoma	6 (6.6%)	4 (8.3%)	2 (4.7%)
Adenocarcinoma with other tissue	18 (19.8%)	13 (27.1%)	5 (11.6%)
**Clinical stage**					
I–II	57 (62.6%)	33 (68.8%)	24 (55.8%)	1.622	0.203
III–IV	34 (37.4%)	15 (31.2%)	19 (44.2%)
**T classification**					
T1–T2	68 (74.7%)	37 (77.1%)	31 (72.1%)	0.299	0.584
T3–T4	23 (25.3%)	11 (22.9%)	12 (21.9%)
**N classification**					
N negative	39 (42.9%)	25 (52.1%)	14 (32.6%)	3.531	**0.028**
N positive	52 (57.1%)	23 (47.9%)	29 (67.4%)
**M classification**					
M0	89 (97.8%)	48 (100%)	41 (95.3%)		0.221
M1	2 (2.2%)	0 (0%)	2 (4.7%)

**Table 4:** Univariate and multivariate Cox regression analysis.

Variables	Univariate Analysis			Multivariate Analysis		
	HR	95% CI	*P* value	HR	95% CI	*P* value
Gender	1.491	0.85–2.618	0.164	1.05	1.017–1.084	0.003
Age	1.039	1.007–1.071	0.017			
Clinical stage (I)			0.017			
Clinical stage (II)	1.116	0.509–2.448	0.784			
Clinical stage (III)	2.545	1.275–5.081	0.008			
Clinical stage (IV)	3.846	0.848–17.442	0.081			
T classification (T1)			0.006			
T classification (T2)	1.883	0.866–4.094	0.11			
T classification (T3)	4.479	1.823–11.006	0.001			
T classification (T4)	3.383	1.252–9.138	0.016			
N classification			0.055			
N classification (1)	1.243	0.625–2.47	0.535			
N classification (2)	2.773	1.298–5.925	0.008			
N classification (3)	1.866	0.737–4.727	0.188			
M classification (M0vsM1)	2.482	0.595–10.357	0.212			
MRPL15 expression	1.895	1.083–3.316	0.025	1.996	1.121–3.554	0.019

## Discussion

Here, we first declared that MRPL15 was up-regulated in tumor tissues in patients with NSCLC *via* multiple cohorts including GEPIA, ONCOMINE and eight GEO series (GSE8569, GSE101929, GSE33532, GSE27262, GSE21933, GSE19804, GSE19188, GSE18842). Moreover, the impact of abnormal MRPL15 expression on different clinical pathological characters was investigated in UALCAN. Kaplan–Meier analysis also demonstrated that high MRPL15 expression was associated with poor OS, PFS, DFS and RFS in NSCLC. Furthermore, using GeneMANIA, functional networks linked to MRPL15 were identified. Additionally, we found that MRPL15 expression was negatively associated with immune scores, stromal scores and several TILs, such as eosinophils and natural killer cells. IHC results further validated the high MRPL15 expression and its prognostic potential in lung cancer. Our results identify a possible biomarker for NSCLC and provide functional mechanisms to be further explored.

MRPL15 is a member of mitochondrial ribosomal proteins (MRPs) and it is connected to OXPHOS. MRPL15 has not been widely studied, but various MRPs play a prognosis and diagnosis role in multiple cancers. Here, we are the first to report that high MRPL15 expression indicates poor prognosis in NSCLC and reveal potential regulatory networks as well as the negative relationship with immune infiltration.

In our work, higher expression of MRPL15 was observed in pan-cancers tissues than adjacent normal tissues using online database, including LUAD and LUSC. Moreover, in NSCLC, MRPL15 expression was associated with different clinical pathological characters including gender, clinical stage, lymph node status and the TP53 mutation status. Kaplan–Meier Plotter also demonstrated a positive relationship between MRPL15 expression and poor clinical outcomes in NSCLC. IHC results further validated the findings: Firstly, MRPL15 expression is related to lymph node metastasis. Secondly, MRPL15 is overexpressed in lung cancer patients and predicts poor OS. Federica Sotgia et al. presented evidence revealing that MRPL15 has predictive value in breast cancer distant metastasis (19). Jandee Lee et al. illustrated that expression of MRPL44 (components of MRPs) was significantly related to lymph node metastasis and influenced OXPHOS in papillary thyroid cancers (PTCs) (46). Furthermore, reduced mitochondrial respiratory chain activity caused by mtDNA decreased promote metastasis and increased glycolysis in lung cancer cells (47, 48). Therefore, we suppose that MRPL15 might impact OXPHOS function and induce poor prognosis.

To explore the biological function of MRPL15 in NSCLC, we first identified co-expression genes associated with MRPL15 in LUAD and LUSD cohorts. Next, the KEGG pathway analysis was performed. The results showed that MRPL15 co-expressed genes were focused on OXPHOS and pyrimidine metabolism and DNA replication. Enrichment analysis of target gene sets through PPI network identify the significant networks of target kinases HCK and transcription factor ELK1 in LUCD and LUSD. These kinases regulate Fc receptor signaling pathway, immune response-regulating cell surface receptor and PI3K signaling pathway. Actually, HCK overexpression has also been observed in NSCLC (49) and HCK activation has been reported to force the recruitment of immune cells into tumors in many cancers (50, 51), which was consistent with the phenotype of up-regulated MRPL15 in our present study.

The network of transcription factors including ELK1 have been linked to amino acid metabolism, DNA damage response, signal transduction by p53 class mediator resulting in cell cycle arrest and signal transduction involved in mitotic DNA integrity checkpoint. In fact, ELK1 could improve LUAD progression (52). ELK1 was identified as a gene with a likely role in OXPHOS biogenesis similar to MRPL15 (53). Nevertheless, more researches are required to understand the function of MRPL15 in NSCLC. Based on our results and previous researches, we provide promising insights into the molecular mechanisms, which will be useful for clinical applications.

Another significant conclusion of our findings is that MRPL15 showed a negative correlation with immune infiltration in NSCLC (especially in LAUD). The high MRPL15 expression group displayed lower immune score and stromal score than the low MRPL15 expression group. Additionally, the results of HLA family genes are consistent with immune score and stromal score. TILs were associated with a positive clinical outcome in several cancers, including lung cancer (54, 55). MRPL15 also showed a negative correlation with immune cells, such as B cell, NK cell and eosinophils in NSCLC. Thus, this data provides evidence for the impact of MRPL15 on immune infiltration in NSCLC.

In conclusion, our study has identified MRPL15 as a novel predictor that contributes to poor prognosis for NSCLC. Furthermore, several kinases and transcription factors related to MRPL15 were recognized, indicating MRPL15 will be a therapeutic target. Overall, MRPL15 may be an attractive prognostic predictor for NSCLC and worthy to be deeply explored in NSCLC.

## Data Availability Statement

The original contributions presented in the study are included in the article/**Supplementary Material**. Further inquiries can be directed to the corresponding authors.

## Ethics Statement

The studies involving human participants were reviewed and approved by The Ethics Committee of Shanghai Outdo Biotech Co., Ltd. Written informed consent for participation was not required for this study in accordance with the national legislation and the institutional requirements.

## Author Contributions

YanZ, YS, and LX participated in data analysis and wrote the manuscript, and they contributed equally to this work. YulZ and XC participated in verification and visualization of the manuscript. YW and NY contributed to carrying out additional analyses. YunZ and FZ conceived the idea and provided guidance. All authors contributed to the article and approved the submitted version.

## Funding

This research was funded by the National Natural Science Foundation of China (81472799).

## Conflict of Interest

The authors declare that the research was conducted in the absence of any commercial or financial relationships that could be construed as a potential conflict of interest.

## Supplementary Material

Supplementary Figure 1 Disease-free survival (DFS) of MRPL15 in lung cancer cohort from GEPIA.

Supplementary Figure 2 PPI network of kinases HCK-target networks in LUSC (GeneMANIA). PPI network and functional analysis indicating the gene set that was enriched in the target network of HCK. The network nodes’ colors represent the biological functions of the enrichment genes.

Supplementary Figure 3 PPI network of transcription factor ELK1-target networks in LUSC (GeneMANIA). PPI network and functional analysis indicating the gene set that was enriched in the target network of ELK1-target. The network nodes’ colors represent the biological functions of the enrichment genes.

Supplementary Figure 4 The expression of HLA family genes in high and low expression MRPL15 groups in NSCLC: **(A)** LUAD and **(B)** LUSC (P < 0.05).
