# Supplementary figures and images for: Prognostic Value and Related Regulatory Networks of MRPL15 in Non-Small-Cell Lung Cancer
Source: Front Oncol. 2021 May 7;11:656172. doi: 10.3389/fonc.2021.656172 (PMC8138120; doi:10.3389/fonc.2021.656172)

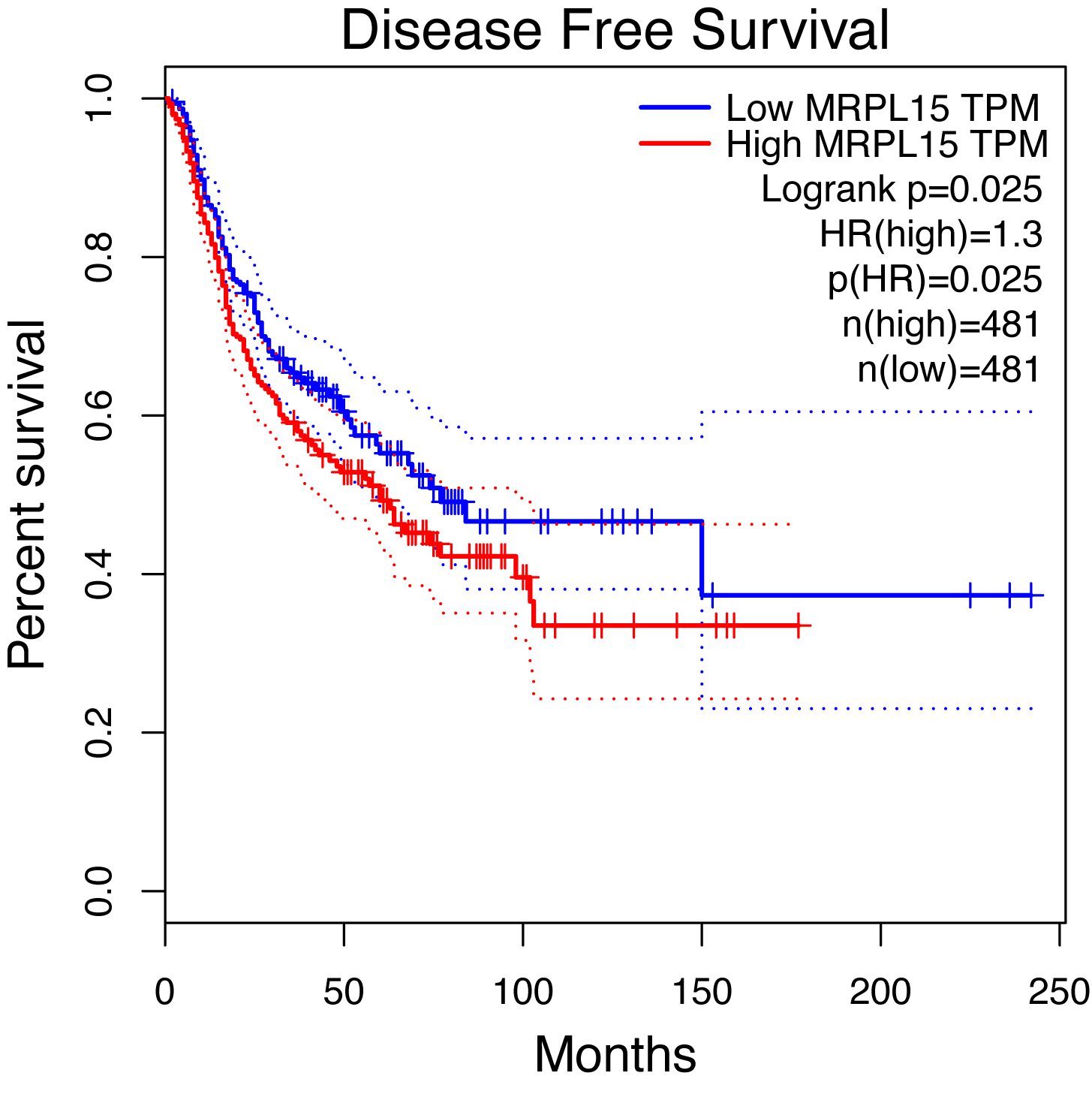

Supplement: Supplementary Figure 1 — Disease-free survival (DFS) of MRPL15 in lung cancer cohort from GEPIA. [file Image_1.tif]

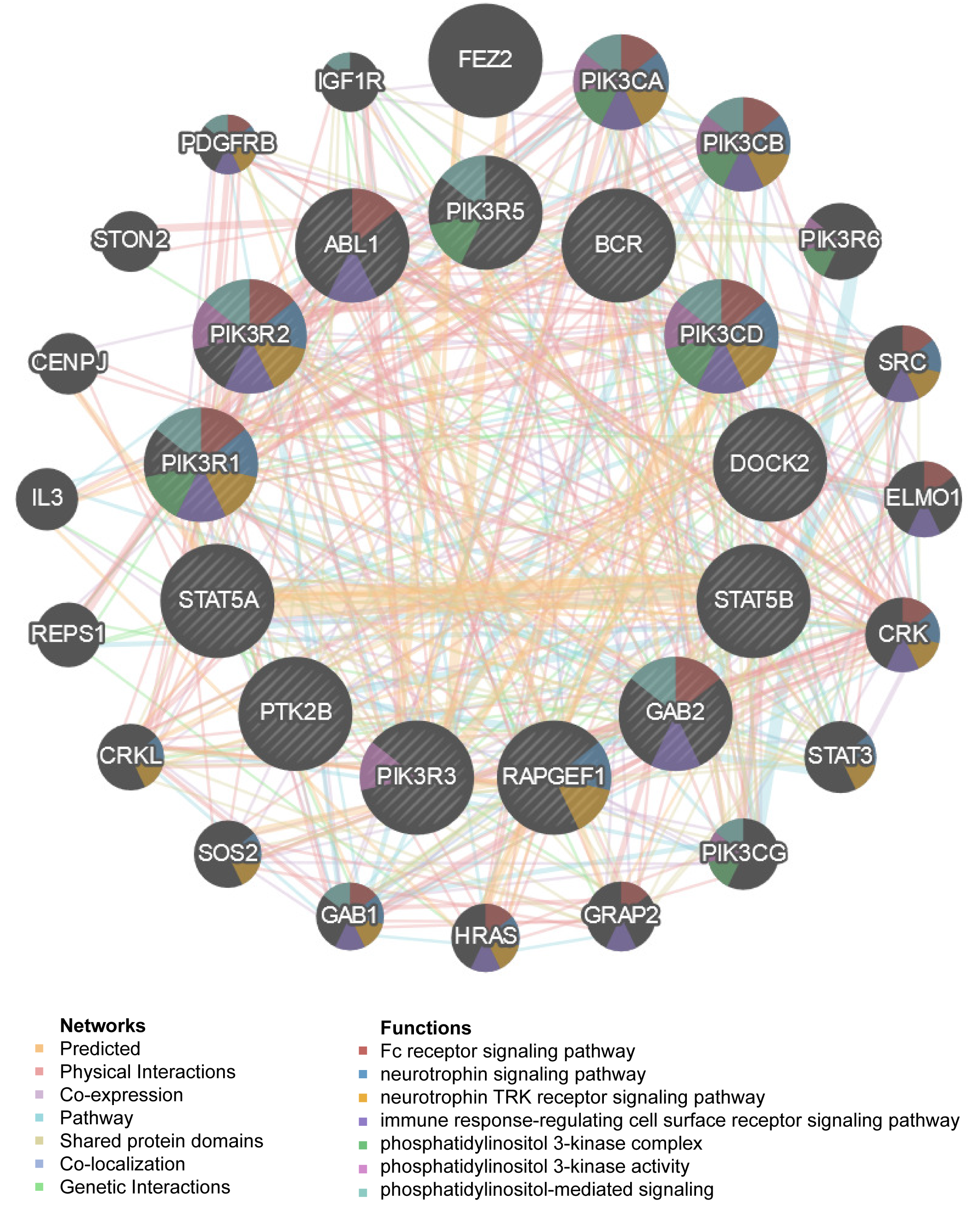

Supplement: Supplementary Figure 2 — PPI network of kinases HCK-target networks in LUSC (GeneMANIA). PPI network and functional analysis indicating the gene set that was enriched in the target network of HCK. The network nodes’ colors represent the biological functions of the enrichment genes. [file Image_2.tif]

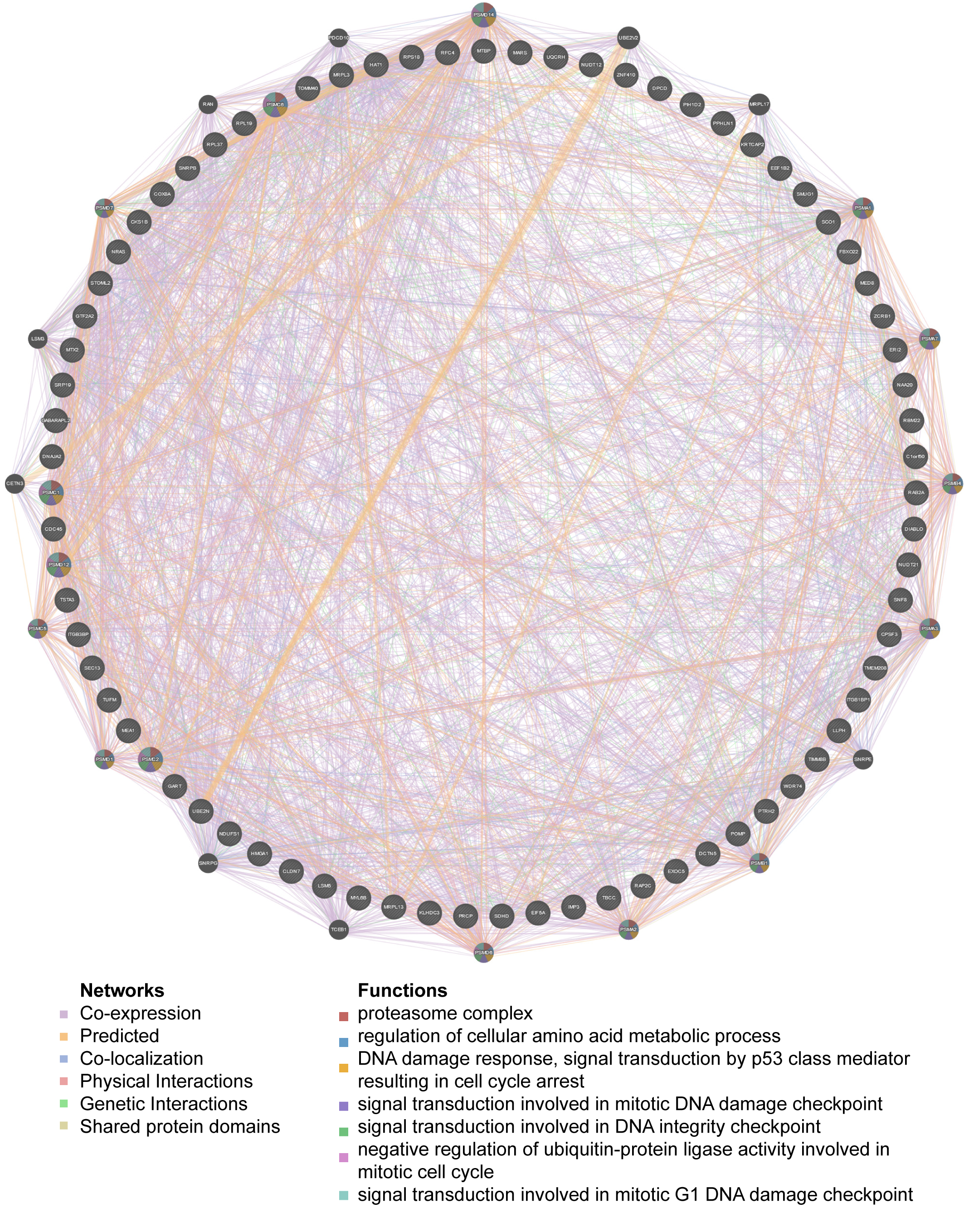

Supplement: Supplementary Figure 3 — PPI network of transcription factor ELK1-target networks in LUSC (GeneMANIA). PPI network and functional analysis indicating the gene set that was enriched in the target network of ELK1-target. The network nodes’ colors represent the biological functions of the enrichment genes. [file Image_3.tif]

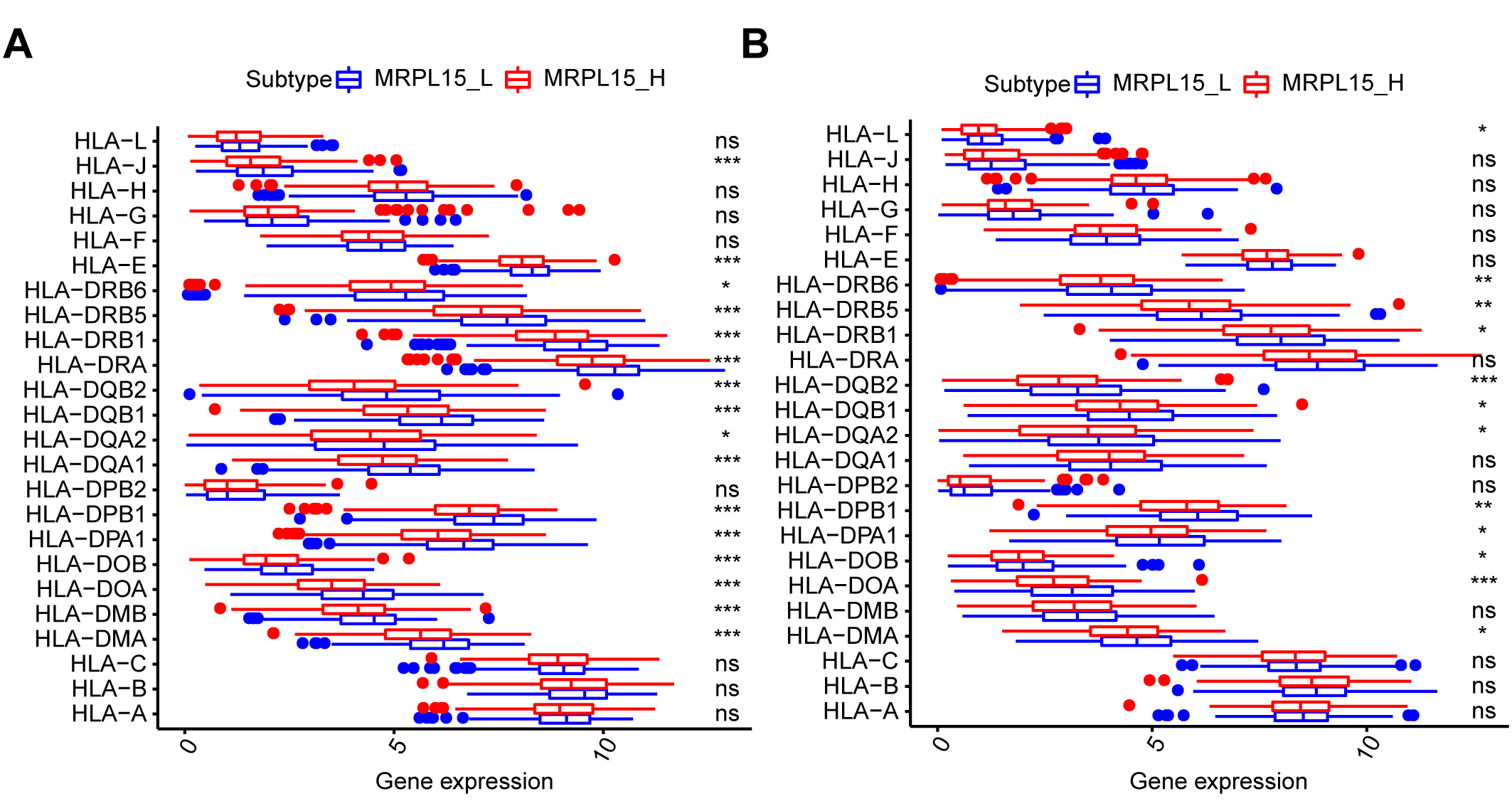

Supplement: Supplementary Figure 4 — The expression of HLA family genes in high and low expression MRPL15 groups in NSCLC: (A) LUAD and (B) LUSC (P < 0.05). [file Image_4.tif]
