# Supplementary material for: Prognostic Value and Related Regulatory Networks of MRPL15 in Non-Small-Cell Lung Cancer
Source: Front Oncol. 2021 May 7;11:656172. doi: 10.3389/fonc.2021.656172 (PMC8138120; doi:10.3389/fonc.2021.656172)
Supplement: Supplementary file 5 [file Table_1.docx]

# SUPPLEMENTARY TABLES

**Supplementary Table S1.** Significantly enriched kinase-target networks of MRPL15 in LUAD (LinkedOmics).

| **Geneset** |  | **LeadingEdgeGene** |
| --- | --- | --- |
| Kinase_HCK |  | ABL1;BCR;DOCK2;ELMO1;GAB2;PIK3CD;PIK3CG;PIK3R1;PIK3R3;PIK3R5;PTK2B;RAPGEF1;STAT5A;STAT5B |
| Kinase_PLK1 |  | ANAPC7;BIRC5;BUB1B;CCNB1;CDC25A;CDC25C;CDC6;CENPQ;CEP55;CHEK2;ERCC6L;ESPL1;FADD;FBXO43;FBXO5;FOXM1;GTSE1;HSF1;KIF2C;NPM1;PINX1;PKMYT1;PRC1;RACGAP1;RAD51;RAN;RUVBL1;SRI;STIL;SUGT1;TERF1;TOP2A;YY1 |
| Kinase_AURKB |  | AURKB;BIRC5;CCDC86;CDCA2;CDCA5;CDCA8;CENPA;CHMP4C;CKAP2;DDX52;DSN1;H3F3A;HIST1H3B;HIST1H3C;HMGN2;HSP90AB1;KIF23;KIF2C;KIF4A;KRT8;MPHOSPH10;MYL12B;NDC80;NUSAP1;PLK1;PPHLN1;PRKDC;RACGAP1;RBM3;RBMX;RPL21;RPL8;RPS10;SHCBP1;YY1 |
| Kinase_PLK3 |  | CALU;CCNB1;CDC25A;CDC25C;CHEK2;HSP90AB1;NPM1;TOP2A;VRK1;YWHAE |
| Kinase_CD1 |  | ABI1;ANAPC11;BIRC5;BLM;BUB1;BUB1B;CCNB1;CDC20;CDC25A;CDC25C;CDC7;CDCA5;CDK7;CENPA;CEP55;CHEK1;CKAP2;CREM;CSNK2B;CUEDC2;DLGAP5;DNM1L;DTL;DUT;E2F1;ECT2;EEF1D;EIF4EBP1;ERCC6L;ESPL1;EZH2;FANCG;FBXO43;FEN1;FOXM1;GMPS;HMGA1;HNRNPK;KIF11;KIF20B;KIF22;KIF2C;KRT8;LDHA;LMNB1;MAPK6;MCM7;MCTS1;NCAPG;NME1;NME2;NOLC1;NPM1;NUSAP1;PAICS;PBK;PPP1CA;PRC1;PRDX1;RCC1;RFC2;RFC3;RFC4;RFC5;RPS3;RRM2;SCML2;SLBP;SPAG5;SSR1;STMN1;TERF1;TK1;TMPO;TOP2A;TPX2;UBE2A;UBXN2B;UGDH;UHRF1;UNG;USP14;VCPIP1;XPO1;ZC3HC1 |

**Abbreviations:** LeadingEdgeNum, the number of leading edge genes; FDR, false discovery rate from Benjamini and Hochberg from gene set enrichment analysis.

**Supplementary Table S2.** Significantly enriched kinase-target networks of MRPL15 in LUSC (LinkedOmics).

| **Geneset** |  | **LeadingEdgeGene** |
| --- | --- | --- |
| Kinase_MAPK7 |  | CAD;DAPK1;ELK4;ETS1;GJA1;MAP2K5;MAPK7;MEF2A;MEF2C;NFKB1;PML;RPS6KA2;RPS6KA6;RPTOR;RUNX1;STAT3;TSC2 |
| Kinase_HCK |  | ABL1;BCR;DOCK2;GAB2;PIK3CD;PIK3R1;PIK3R2;PIK3R3;PIK3R5;PTK2B;RAPGEF1;STAT5A;STAT5B |
| Kinase_PRKAA2 |  | ACACA;ACACB;EEF2K;FOXO3;HDAC5;IKBKB;MTOR;PLD1;PPP1R12C;RPTOR;TSC2 |
| Kinase_MAPK10 |  | ATN1;FOSL2;FOXO1;FOXO3;FOXO4;JUN;MADD;NFATC1;NFATC2 |
| Kinase_MAPK3 |  | AR;ARHGEF2;BAZ1B;BCL2;BCL2L11;BCL6;CAD;CALD1;CAPN2;CIC;CIITA;CREBBP;DAPK1;EGFR;EIF4G1;ETS1;ETV6;EWSR1;EXOC7;FBXW7;FOXO3;GAB2;GATA6;GJA1;GSK3B;GTF2I;HDAC6;IRS1;ITGB4;JUN;LIFR;LRP6;MAPK7;MED1;MITF;MKNK2;MYLK;NCOA1;NCOA2;NFATC1;NFKB1;NUP153;NUP214;NUP50;PGR;PIP5K1C;PML;ROCK1;ROCK2;RPS6KA2;RPS6KA6;RPTOR;RUNX1;RUNX2;RXRA;SH2B1;SMAD3;SMAD9;SP1;SPHK2;SREBF2;STAT3;STAT5A;TAL1;THRB;TSC2;WASF2 |

**Abbreviations:** LeadingEdgeNum, the number of leading edge genes; FDR, false discovery rate from Benjamini and Hochberg from gene set enrichment analysis (GSEA).

**Supplementary Table S3.** Significantly enriched miRNA-target networks of MRPL15 in LUAD (LinkedOmics).

| **Geneset** |  | | **LeadingEdgeGene** |
| --- | --- | --- | --- |
| ATGTACA,MIR-493 |  | AFF3;AHDC1;ANKRD17;ANKRD50;APLP2;AR;ARID1A;ARID1B;BAZ2A;BCL2;BCOR;BHLHE22;BHLHE41;BTAF1;CAPN3;CDC14A;CDH11;CELF2;CITED2;CLASP1;COBLL1;CREBBP;CTDSPL;CTNND1;DAB2IP;DACH1;DDX3X;DIP2C;DKK2;DLG2;DNAJC13;DOCK9;EIF4ENIF1;ESRRG;FBRS;FNIP1;FOXO1;FZD4;GAB1;GBF1;GPC4;HEMGN;HIPK1;HIVEP2;ITSN2;JUN;KDM6A;KIAA2026;KIF1B;KLF3;LCA5;LRIG1;MADD;MAML3;MBD5;MBNL2;MED13L;MED26;MEF2C;MFSD6;NBEA;NCOA1;NDST1;NLGN3;NRXN3;OSBPL6;PCDH18;PDPK1;PDZRN3;PHF12;PHF2;PIAS1;PIK3R1;PIKFYVE;PPP1R10;PREX1;PRPF38B;RALGPS1;RBBP6;RIMS3;RNF38;RPS6KA5;SH2B3;SH2D3C;SIN3A;SIPA1L2;SIPA1L3;SLC22A23;SMG1;SP4;SPAG8;SPEN;SPRED2;SSH2;SVIL;SYNE1;TAB3;TAOK3;TBC1D4;TJP1;TLE4;TNRC6B;TRAM2;TTN;VAMP2;VGLL4;VPS13D;WDFY3;WNT5A;ZFC3H1;ZFX;ZMIZ1;ZNF362;ZNF385B;ZNF711 | |
| GTGCAAA,MIR-507 |  | ANKRD12;ANO4;BACH2;BICD2;BMP4;BRD4;CHD9;CPEB4;DAB2IP;DLG3;DLL1;DNAJC13;DYRK1A;EP300;ESRRG;FAM43A;FBXW7;IQSEC2;MAB21L1;MAF;MAP3K3;MED13L;MEIS1;NCOA1;NELL2;PARP16;PAX6;PBRM1;PCDH19;PCF11;PJA2;PRKCE;RAPH1;RBMS3;REV3L;RSBN1;RUNX1;SECISBP2L;SEMA6D;SLC24A4;SLTM;SORBS2;SORCS1;STAT5B;THRA;TP63;TSPYL4;UNKL;USP47;ZER1;ZFX | |
| GTGTGAG,MIR-342 |  | AGPAT4;ARHGEF37;ARID1B;BMPR2;BTBD7;CTNND1;DDX3X;EDA;EP300;FRMD4A;INO80;KLF13;MEIS1;MINK1;NBEA;PDGFRA;PPM1F;RASA1;RPS6KA5;SOX6;TRIOBP;UBN1;ZNF462 | |
| TTTGCAC,MIR-19A, MIR-19B |  | ABR;ADCY7;ADCY9;AFF1;ANKRD12;AP1G1;ARC;ARGLU1;ARHGAP1;ARHGEF12;ARID4B;ARRDC4;ATP11A;ATRX;ATXN1;BACE1;BMPR2;BNC2;BPTF;BRWD1;BSN;BTAF1;BTBD7;C3orf70;CACNA1C;CAMSAP1;CBX7;CC2D1A;CCND2;CDK13;CDK19;CEP192;CEP350;CNOT4;CNTFR;COL19A1;CPEB4;CREBL2;CYLD;DDX3X;DDX6;DICER1;DLC1;DNAJC16;ENPP5;EPS15;ERBB4;ETV1;EVI5L;FAM43A;FEM1C;FOXF2;FOXP1;FZD8;GRIN2A;GRM7;HIPK1;HLF;IGF2R;IGSF3;INHBB;INO80;ITPR1;ITSN1;KCNA4;KCNS2;KDM2A;KIF3A;KLF13;KLHL20;LBH;LRIG1;LRRK1;MACF1;MAGI2;MAP3K12;MAP3K14;MBD6;MBNL2;MECP2;MED13L;MED26;MEF2D;MFSD6;MINK1;MLLT6;NAPB;NBEA;NHS;OGT;OLFM1;PARM1;PCDH10;PCDHA10;PCDHA12;PCDHA3;PCDHAC2;PDE5A;PDE7B;PHF12;PIK3R3;PLXNC1;PPARA;PRICKLE2;PRRT3;PTK2B;PTPRG;RAI2;RALGPS1;RAPGEF4;RBMS3;RFX1;RGL1;RNF111;RNF165;RNF38;ROBO2;RTN1;RUNX3;RXRA;S1PR1;SCARF1;SDC1;SGK1;SH3D19;SHANK2;SLC24A3;SLC24A4;SLC9A1;SLC9A6;SMARCA2;SOX5;SOX6;SPEN;SRGAP3;ST3GAL5;STAT5B;TBC1D8;TESK2;TGFBR2;TGOLN2;TLN2;TMEM63B;TNRC6A;TNRC6B;TRAK2;TRIM33;TSC1;TSHZ3;UBL3;UCP3;VGLL4;WDFY3;WDR47;ZBTB4;ZDHHC7;ZEB2;ZFPM2;ZFYVE26;ZFYVE9;ZNF609;ZNF711 | |
| GCACCTT,MIR-18A, MIR-18B |  | ADAMTS13;ALCAM;ANKRD50;ARL15;ASXL2;ATXN1;BHLHE22;BRWD3;CDK19;CLIP3;CREBL2;CRIM1;CTDSPL;DIP2C;EHMT1;EPB41L1;FCHSD2;FNBP1;FRMD4A;GAB1;GIGYF1;IRF2;KDM2A;KLHL20;MAN1A2;MAP7D1;MDGA1;MEF2D;NCOA1;NEDD9;NFAT5;PDZD2;PHF2;PRICKLE2;PSD3;RAB11FIP2;RABGAP1;RUNX1;SMAP2;SON;SORBS2;STK4;TEX2;TNRC6B;TRIB2;TRIM2;TRIOBP;TSHZ3;ZBTB4;ZBTB44;ZBTB47;ZFP36L1;ZNF704 | |

**Abbreviations:** LeadingEdgeNum, the number of leading edge genes; FDR, false discovery rate from Benjamini and Hochberg from gene set enrichment analysis (GSEA).

**Supplementary Table S4.** Significantly enriched miRNA-target networks of MRPL15 in LUSC (LinkedOmics).

| **Geneset** |  | **LeadingEdgeGene** |
| --- | --- | --- |
| TATTATA,MIR-374 |  | ABCA8;ABR;ACTN4;ADAM10;AFF1;AFF4;ARHGAP28;ARHGAP6;ARID2;ATP1A1;ATP8B2;ATXN1;BACE1;BANK1;BICD2;BMP2;BMPER;BSDC1;CBX4;CHST2;CRIM1;CRTC2;CSMD2;CYP26B1;DACH1;DENND4A;DMD;DUSP8;EDAR;EIF3A;EP300;ESRRG;ETV6;EYA1;FAF2;FBXO42;FGFR2;FNDC3B;FOSB;FOXO1;FOXP1;FRMPD4;GATA3;HDHD2;HECTD1;HERC2;HIVEP2;HLF;IQSEC2;KIAA2026;LARP1;LCA5;LRRN1;LTBP1;MECP2;MED13;MEF2D;MEIS1;MEX3B;MEX3C;MMP14;NCOA1;NCOA6;NEO1;NFIX;NIPBL;NLGN1;NOG;NR2F2;NTM;NUAK1;NUMBL;OSBP;PAPPA;PAXIP1;PCDH10;PCGF3;PDE7B;PDPK1;PELI1;PHF21A;PHRF1;PIKFYVE;PKIA;PMEPA1;PRDM1;PRKCD;RAI1;RALGDS;RC3H1;RGS7BP;RNF38;RORB;RSF1;RUNX2;SCN2B;SERTAD2;SETD2;SF1;SMAD6;SOX4;SP1;SPRY2;ST8SIA2;STARD13;SYNE1;TCF12;TEAD3;TLE4;TMEM108;TMEM47;TNRC6A;TXLNB;UBE2H;UST;VAMP2;VEZF1;VWC2;WDR11;WNT5A;WNT5B;ZC3H7B;ZCCHC14;ZCCHC24;ZEB2;ZER1;ZNF236;ZNF423;ZNF711 |
| TGGTGCT,MIR-29A, MIR-29B, MIR-29C |  | ADAM12;ADAM19;ADAMTS13;ADAMTS9;AFF4;AGPAT4;AKAP13;AKT3;AMOT;ANK3;AP4E1;ARNT;ARRDC4;ARVCF;ATG9A;ATP2B4;ATRN;ATXN1;BACH2;BCL11A;BCL7A;BCL9L;BCORL1;BIRC6;BMF;BMP1;BRD4;BRWD1;BRWD3;CA3;CCND2;CD276;CELF6;CHSY1;CLK2;CNR1;COL15A1;COL16A1;COL19A1;COL1A1;COL3A1;COL4A1;COL4A2;COL4A3;COL4A4;COL4A5;COL5A2;COL5A3;COL6A3;COL7A1;COL9A1;CPEB3;CREB5;CTNND1;DAB2IP;DAG1;DCAF7;DDX3X;DGKD;DICER1;DIP2B;DNM3;DNMT3A;ELMO2;EPHA3;FAM131B;FAM13B;FAM193B;FBN1;FBRS;FBXO24;FBXW7;FOXJ2;FRAS1;GAB1;GCC2;GLIS2;HDAC4;HECW1;HIF3A;HMCN1;HNRNPUL1;IFFO1;IGF1;INO80D;IREB2;ISL1;KCNIP2;KDM2A;KDM5C;KIAA0355;KIF3B;KLF12;LAMC1;LDLRAP1;LGI2;LOXL4;LPL;LUZP1;MAFB;MAP4K4;MAPKBP1;MMP2;MTMR4;MYCN;NAP1L3;NAV1;NAV2;NAV3;NCOR2;NDST1;NFAT5;NFATC4;NFIA;NFIX;NKTR;NLGN3;OSBP;OSBPL11;OTUD4;PALM;PAN2;PCDHAC1;PCDHAC2;PCGF3;PDGFB;PDGFRB;PER1;PER3;PGAP1;PHC1;PIK3R1;PIK3R3;POU2F2;PPARD;PRELP;PRICKLE2;RAB40C;RASGRP4;REV3L;RGPD5;RNF165;RYBP;SBF2;SESTD1;SH3BP5L;SH3PXD2A;SHPRH;SHROOM2;SIDT1;SKI;SMPD3;SP1;SPEN;SPPL2B;SPRY1;SPTAN1;SRGAP2;SRGAP3;STARD8;STRN4;STX17;SYNE1;TMEM127;TRIB2;TSPAN4;USP42;WDFY1;WDFY3;XKR4;YPEL2;ZBTB40;ZBTB46;ZBTB47;ZER1;ZFP36;ZFP36L1;ZFX;ZFYVE26;ZNF282;ZNF362;ZNF512B;ZNF609 |
| TGTGTGA,MIR-377 |  | ABCA9;ADARB1;ARID1A;ARID1B;ARID4B;ATXN1;ATXN2L;ATXN7;ATXN7L1;BAZ2A;BAZ2B;C2CD2L;CACNA2D2;CD93;CDON;CELF2;DLGAP4;EGR1;EGR2;EP300;ETS1;FLNC;FOXN3;FZD4;GGA3;H6PD;JAZF1;LAMC1;MACF1;MADD;MAP1A;NBEA;NCOA1;NCOA6;PCDH10;PHF21A;PHF3;PPM1F;PRKD1;PRX;PUM1;RABGAP1;RNF165;RNF38;STK35;STRN4;TEAD1;TRIOBP;TRIP12;XIAP;ZBTB4;ZEB2;ZFP36L1;ZNF462 |
| AGGGCAG,MIR-18A |  | AGPAT4;ARID1A;ATF7;BAHD1;CBX7;CCDC88B;CIT;CITED2;CSNK1D;CTDP1;CYFIP2;DAG1;DLC1;DLEC1;EIF4ENIF1;ENC1;FURIN;GATA2;GLP1R;GYS1;HIC2;HIRA;HMGN5;ITGB3;KDM2A;KHNYN;LRRC8A;MAPK8IP3;MAPKBP1;MXD4;NF2;PAK3;PBRM1;PCDHAC1;PCDHAC2;RNF40;RUNX1;SERTAD2;SETD2;SMPD3;SNX27;ST8SIA2;STAT3;STRADA;TACC1;TET2;TIMP2;TLN2;TRIB2;TRIM25;TTLL3;WASF2;ZBTB4;ZC3H12B;ZCCHC14;ZFYVE1 |
| GCAAAAA,MIR-129 |  | AGAP4;APC;ARNT;ATP2B4;BACH2;BCL6;BMPR2;BPTF;CBX7;CDC42EP3;CELF2;CEP192;CTNND1;DAB2IP;DDX3X;DNAJC13;EFNB2;EIF4G3;EP300;ESRRG;ETS1;ETV1;FAM13B;FBXW7;FNDC5;GABBR2;GFRA2;GLYR1;GRM7;HIC2;ILF3;KDM2A;LMNA;MED13L;MED26;MEIS1;MEIS2;NIPBL;NR2C2;NR3C2;NUFIP2;PCDH7;PDLIM5;PDZRN4;PHF12;PHF21A;PIK3R1;PLCG1;PRDM1;PRKCB;PSD3;RBMS3;RNF165;RUNX1;RUNX1T1;SBK1;SEC14L1;SLC25A27;SLTM;STAT5B;STOX2;SUN2;TP53INP1;TRIO;TSHZ1;USP6;XKR4;ZFP36L1;ZFX;ZNF608;ZNF609 |

**Abbreviations:** LeadingEdgeNum, the number of leading edge genes; FDR, false discovery rate from Benjamini and Hochberg from gene set enrichment analysis (GSEA).

**Supplementary Table S5.** Significantly enriched transcription factor-target networks of MRPL15 in LUAD (LinkedOmics).

| **Geneset** |  | **LeadingEdgeGene** |
| --- | --- | --- |
| V$ELK1_02 |  | AP2S1;ARL5B;CALU;CDC45;CKS1B;CLDN7;COX8A;CPSF3;DCTN5;DDOST;DIABLO;DNAJA2;DPCD;EEF1B2;EIF5A;FANCD2;FBXO22;GART;GTF2A2;HAT1;HMGA1;IMMT;IPO4;ITGB1BP1;ITGB3BP;KRTCAP2;LLPH;LSM5;MARS;MEA1;MED8;MRPL13;MRPL3;MTBP;MTX2;MYL6B;NAA20;NDUFS1;NRAS;POMP;PPHLN1;PSMC1;PSMC2;PSMC6;PSMD12;PTRH2;RAB2A;RFC4;RPL19;RPL37;RPS18;SCO1;SDHD;SEC13;SMS;SMUG1;SNF8;SNRPB;SRP19;STOML2;TBCC;TIMM8B;TMEM208;TOMM40;TSTA3;TUFM;UBAC1;UBE2N;UQCRH;WDR74;ZCRB1;ZNF410 |
| SCGGAAGY_V$ELK1_02 |  | AAGAB;ACBD5;ACTR6;AIP;ALDOA;AMZ2;AP2S1;AP4M1;APTX;ARFIP2;ARL5B;ARPC2;ARPC4;ATAD1;ATG3;ATG5;ATP6V1H;AURKA;BANF1;BLM;BLZF1;BZW2;C12orf57;C12orf66;C14orf119;C17orf80;C19orf47;C1QBP;C1orf122;C2orf49;C3orf38;C9orf40;CALU;CARS;CBX8;CCT7;CDC123;CDC45;CDCA3;CENPO;CEP55;CETN3;CHORDC1;CHUK;CIAO1;CKS1B;CLDN7;COMMD5;COPE;COPS3;COPS7A;COX15;COX17;COX5B;COX6A1;COX6B1;COX8A;CPSF3;CSNK2B;CSTF1;CSTF3;CUTC;CWC15;CYB5R4;DCTN5;DDIT3;DDOST;DDX1;DDX47;DDX49;DDX50;DDX55;DGUOK;DHX15;DIABLO;DNAJA1;DNTTIP1;DPCD;DPH2;DPM1;DR1;EBNA1BP2;ECD;EEF1B2;EFTUD2;EIF2B2;EIF2S1;EIF3H;EIF4A1;EIF5A;ELP2;EME1;EMG1;ERCC1;ERCC3;ERH;EXOSC3;EXOSC5;FAM104A;FAM200A;FANCD2;FBXL6;FBXO22;FIBP;FKBPL;FOXRED1;GAPDH;GAR1;GART;GNAI3;GRPEL2;GTF2A2;HAT1;HCCS;HSP90AB1;HSPA4;IPO4;ITGB1BP1;ITPA;JAGN1;KIF4A;KRR1;KRTCAP2;KTI12;LLPH;LSM4;LSM5;LZIC;MAD2L1BP;MAP4K3;MAPRE1;MARS;MASTL;MCM7;MCRS1;MCTS1;MEA1;MED30;MED8;METAP2;METTL5;METTL6;MFN1;MFSD5;MOCS3;MORN2;MPDU1;MRPL27;MRPL3;MRPL33;MRPL40;MRPL43;MRPL52;MRPS10;MRPS18A;MRPS21;MTIF2;MTX2;MYL6B;MYO19;NAA20;NARS;NDUFS1;NECAP1;NEDD8;NIF3L1;NOL7;NOSIP;NPRL2;NRAS;NSUN2;NUDT5;NUDT6;NUP107;NUP155;NUP37;NUTF2;OTUB1;OVCA2;PABPC1;PARL;PARS2;PCBP1;PCBP2;PCGF1;PDAP1;PDCD6;PDZD11;PEX2;PFDN6;PGAM5;PGM3;PHB2;PHF20L1;PHF23;PHF5A;PIGC;PIGW;PITX2;PLK4;PLP2;PMM2;PMPCB;PNPLA8;POLDIP2;POLR1C;POLR2F;POLR2H;POLR2K;POMP;PPHLN1;PPIL1;PPIL3;PPP1R11;PPP2R3C;PPP4C;PRELID1;PRKAG1;PRPF19;PSMA4;PSMA5;PSMA6;PSMB1;PSMB7;PSMC1;PSMC2;PSMC4;PSMD12;PSMD13;PSME3;PTPN2;PTRH2;PUF60;PUS1;PYROXD1;R3HDM1;RAB2A;RAD23A;RB1CC1;RBM45;RFC2;RFC4;RNF181;RNF2;RNF41;ROMO1;RPE;RPF1;RPL19;RPL26;RPL27;RPL31;RPL32;RPL36AL;RPL37;RPL37A;RPL6;RPLP2;RPS14;RPS18;RPS19BP1;RPS25;RPS3;RPS3A;RPS5;RPS6;RRP15;RRS1;RWDD2A;SAR1A;SCFD1;SCO1;SDF2;SDHAF2;SEC11A;SEC13;SF3B4;SKP2;SLC25A17;SLC25A5;SMUG1;SND1;SNF8;SNRNP27;SNRPB;SNRPE;SRP14;SRP19;SSBP1;STOML2;SUGT1;SUMO1;TAF10;TAF11;TBCC;TBCE;TBP;TFB2M;TGDS;TGS1;THUMPD3;TIMM10;TIPRL;TMCO1;TMEM101;TMEM199;TMEM208;TMEM33;TMEM68;TOMM20;TOMM22;TOMM40;TPM3;TPX2;TRAPPC4;TRMT112;TRMT6;TRPT1;TSTA3;TUFM;TXNDC12;UBA2;UBE2F;UBE2N;UBE2V2;UBL5;UFC1;UNC50;UQCRH;URM1;USF1;UXT;VCPIP1;WDR74;WRAP53;WTAP;XPO5;XRCC4;YARS;YKT6;YME1L1;YTHDF3;YWHAE;ZBTB8OS;ZCCHC7;ZCCHC9;ZCRB1;ZNF410 |
| GGAANCGGAANY_UNKNOWN |  | ATP6V1E1;BANF1;COX6B1;COX7A2;CSNK2B;DDX55;DPM1;EBNA1BP2;EIF2S1;EIF2S3;EIF3H;EIF3K;FARSA;GLRX5;MED8;MRPL21;MRPL43;MRPS18A;MRPS21;MRPS23;PDAP1;POMP;PSMB4;RARS;RNF25;RPL38;RUVBL2;SDF2;SEC11A;SEC61G;SMUG1;SNRPE;TAF10;TFB2M;THUMPD3;TIMM8A;TMCO1;UBA52;UBL5 |
| TMTCGCGANR_UNKNOWN |  | BZW1;CCT8;CDC5L;CHUK;COQ9;COX11;COX7B;DCTN2;DENR;DGUOK;HNRNPK;MARCH7;MDH2;NAA38;NDUFA11;NOL11;NUDT2;PLOD2;POLR1C;PRDX1;PRDX4;PSMB2;PUF60;RPL10A;RPL12;RPL17;RPL26;RPS15A;RPS19;RPS6;RPS7;SAP18;SKA3;SLC25A11;STYXL1;SUV39H1;THAP1;TMCO1;TMEM183A;TROAP;TSR1;UBE2D3;VDAC3;WDR77 |
| V$DBP_Q6 |  | AFF3;ANK2;ANKS1B;ARID4A;AUTS2;BHLHE41;BTK;CA3;CCDC30;CD93;CRY2;DAB2IP;DCHS1;DDX17;DLG2;DMD;ERG;FAM13C;FCGBP;GFRA1;GIPR;GPLD1;IGFALS;ITGA10;ITPKB;ITPR3;ITSN2;JPH4;KCNQ1;KIAA0355;KLF3;LMO3;MAP4K5;MBNL2;MICALL2;MITF;MYLK;NAP1L5;NFAT5;NFIA;NFIX;ORMDL3;OTC;PCDH11X;PCDH17;PDZD2;PLA2G1B;PLXNA2;PTCH1;PTPRG;PURA;RERE;ROS1;SCUBE1;SEMA6A;SFTPC;SMARCA2;SORBS2;STX17;SVIL;TAPBP;TCF21;TLE4;TSHZ3;TSSK3;VSIG2;ZEB2;ZNF385B |

**Abbreviations:** LeadingEdgeNum, the number of leading edge genes; FDR, false discovery rate from Benjamini and Hochberg from gene set enrichment analysis (GSEA). V$, the annotation found in Molecular Signatures Database (MSigDB) for tran-scription factors (TF).

**Supplementary Table S6.** Significantly enriched transcription factor-target networks of MRPL15 in LUSC (LinkedOmics).

| **Geneset** |  | **LeadingEdgeGene** |
| --- | --- | --- |
| GGAANCGGAANY_UNKNOWN |  | ATP6V1D;ATP6V1E1;BANF1;BCDIN3D;COMMD6;COX6B1;COX7A2;CSNK2B;DPM1;EBNA1BP2;EIF2S1;EIF3H;EIF3K;GIN1;GLRX5;MED8;MRPL21;MRPL43;MRPS18A;MRPS21;MRPS23;POMP;PSMB4;RARS;RPL38;RUVBL2;SDF2;SEC11A;SEC61G;SMUG1;SNRPE;SRP54;TAF10;TFB2M;THUMPD3;TIMM8A;TMCO1;UBA52;UBL5 |
| SCGGAAGY_V$ELK1_02 |  | AAGAB;ACTR6;ACYP1;AIP;AKTIP;ALDOA;ALKBH1;AMZ2;APEH;APTX;ARFIP1;ARPC4;ATG5;ATP6V1D;ATP6V1H;AURKA;BANF1;BCDIN3D;BLZF1;BZW2;C12orf57;C12orf66;C14orf119;C1QBP;C2orf49;C3orf38;C9orf40;CCT7;CDC123;CDC45;CDCA3;CDX2;CEP55;CETN3;CHORDC1;CHUK;CIAO1;CKS1B;CLDN7;CNOT10;COMMD5;COMMD6;COPE;COPS3;COX15;COX17;COX5B;COX6A1;COX6B1;COX8A;CPSF3;CSNK2B;CSTF1;CUTC;CWC15;CYB5R4;DCTN5;DDIT3;DDX47;DDX50;DGUOK;DIABLO;DLST;DNTTIP1;DPCD;DPM1;EBNA1BP2;ECD;EEF1B2;EIF2AK1;EIF2B2;EIF2S1;EIF3H;EIF4A1;EIF5A;EMG1;ERH;ERI2;EXOC5;EXOSC3;EXOSC5;FAM192A;FBXO22;FBXO8;FIBP;FKBPL;FOXRED1;GABARAPL2;GAPDH;GART;GMPR2;GRPEL2;GTF2A2;HAT1;HCCS;HSP90AB1;HSPA4;ITGB1BP1;ITPA;JAGN1;KAT5;KLHDC3;KRR1;KRTCAP2;KTI12;LLPH;LSM4;LSM5;LYRM1;LZIC;MAD2L1BP;MAP4K3;MARS;MASTL;MCRS1;MCTS1;MEA1;MED30;MED8;METAP2;METTL5;METTL6;MGAT2;MIER1;MORN2;MPDU1;MRPL27;MRPL3;MRPL33;MRPL40;MRPL43;MRPL52;MRPS10;MRPS18A;MRPS21;MTIF2;MTX2;NAA20;NDUFS1;NECAP1;NEDD8;NIF3L1;NOL7;NOSIP;NPRL2;NRAS;NTHL1;NUDT12;NUDT21;NUDT5;NUDT6;NUP107;NUP37;NUTF2;NXT2;OTUB1;OVCA2;PABPC1;PAIP2;PARL;PCBP2;PCGF1;PDCD6;PDZD11;PEX2;PFDN6;PGAM5;PHB2;PHF5A;PIGC;PLP2;PMM2;PMPCB;PNPLA8;POLDIP2;POLR1C;POLR2F;POLR2H;POLR2K;POMP;PPHLN1;PPIL1;PPIL3;PPP2R3C;PPP4C;PRDX5;PRELID1;PRKAG1;PSMA4;PSMA5;PSMA6;PSMB1;PSMB7;PSMC1;PSMC2;PSMD12;PSMD13;PTRH2;PUF60;PYROXD1;RAB2A;RB1CC1;RBM22;RBM45;RFC2;RFC4;RFESD;RNF13;RNF181;RNF2;RNF41;ROMO1;RPE;RPF1;RPL19;RPL26;RPL27;RPL31;RPL32;RPL36AL;RPL37;RPL37A;RPL6;RPLP2;RPS14;RPS18;RPS19BP1;RPS25;RPS3;RPS3A;RPS5;RPS6;RRS1;SAR1A;SCFD1;SCO1;SDF2;SDHAF2;SEC11A;SEC13;SEPT10;SLC25A17;SLC25A38;SLC25A4;SLC25A5;SMUG1;SNF8;SNRPB;SNRPE;SPG21;SRP14;SRP19;SRP54;SSBP1;SSU72;STOML2;SUGT1;SUMO1;TAF10;TAF11;TAX1BP1;TBCC;TBCE;TFB2M;TGDS;TGS1;THAP11;THG1L;THUMPD3;TIMM10;TIPRL;TMBIM4;TMCO1;TMEM101;TMEM186;TMEM199;TMEM208;TMEM62;TMEM68;TMX1;TOMM20;TOMM22;TPM3;TPX2;TRAPPC1;TRAPPC4;TRMT112;TRPT1;TSTA3;TUFM;TXNDC12;UBA2;UBE2E3;UBE2F;UBE2N;UBE2V2;UBL5;UBLCP1;UFC1;UNC50;UQCRH;URM1;UXT;VCPIP1;WDR74;WTAP;XRCC4;YIPF5;YTHDF3;YWHAE;ZBTB8OS;ZCCHC7;ZCCHC9;ZCRB1;ZNF35;ZNF410 |
| TMTCGCGANR_UNKNOWN |  | BZW1;C5orf24;CCT8;CDC5L;CHUK;CHURC1;COQ9;COX11;COX7B;DCTN2;DENR;DGUOK;EIF5;FGFR1OP2;HSPA4;LRRC28;MAPK14;MARCH7;MDH2;NAA38;NDUFA11;NOL11;NUDT2;PLOD2;POLR1C;PRDX1;PRDX4;PSMB2;PUF60;RAB33B;RNF167;RPL10A;RPL12;RPL17;RPL26;RPS15A;RPS19;RPS6;RPS7;RRAGA;SAP18;SEPT7;SKA3;SLC25A11;SLC25A4;SSR4;STYXL1;SUV39H1;THAP1;TMCO1;TMEM183A;TROAP;UBE2D3;VDAC3;WDR77 |
| V$ELK1_02 |  | C1orf50;CDC45;CKS1B;CLDN7;COX8A;CPSF3;DCTN5;DIABLO;DNAJA2;DPCD;EEF1B2;EIF5A;ERI2;EXOC5;FBXO22;GABARAPL2;GART;GTF2A2;HAT1;HMGA1;IMP3;ITGB1BP1;ITGB3BP;KLHDC3;KRTCAP2;LLPH;LSM5;MARS;MEA1;MED8;MRPL13;MRPL3;MTBP;MTX2;MYL6B;NAA20;NDUFS1;NRAS;NUDT12;NUDT21;PIH1D2;POMP;PPHLN1;PRCP;PSMC1;PSMC2;PSMC6;PSMD12;PTRH2;RAB2A;RAP2C;RBM22;RFC4;RPL19;RPL37;RPS18;SCO1;SDHD;SEC13;SMUG1;SNF8;SNRPB;SRP19;STOML2;TBCC;TIMM8B;TMEM208;TOMM40;TSTA3;TUFM;UBE2N;UQCRH;WDR74;ZCRB1;ZNF410 |
| AAAYWAACM_V$HFH4_01 |  | ADAMTS14;ANKRD11;ARHGAP30;ARHGEF2;ARID1B;ASXL1;ATF7IP;ATOH8;ATP2A2;BCL6;BCOR;C1QTNF3;CALD1;CDK11A;CDK11B;CRIM1;CRY2;DMD;DOCK3;EGR2;EIF4G1;ENPP2;EPB41L3;EVL;FGF17;FOSB;FOXJ3;FSTL1;GATA6;GFRA1;GOLGA1;GRIK2;HMCN1;HOXA6;IKZF3;INMT;IRF4;IRS1;ITGBL1;KLF12;KLF7;KLHL3;MAML2;MAP3K3;MCC;MEF2C;MGLL;MITF;NALCN;NAV3;NEO1;NFIX;NIPBL;NLGN3;NR2F2;NTN1;ODF3L1;PBX1;PIK3C2A;PRDM1;PRX;RBP3;REPS2;SDK2;SIN3A;SLIT3;SMARCA2;SPATA18;SPON1;STARD13;STX16;TBX3;TCF4;TJP1;TLE3;TRIM8;VASN |

**Abbreviations:** LeadingEdgeNum, the number of leading edge genes; FDR, false discovery rate from Benjamini and Hochberg from gene set enrichment analysis (GSEA). V$, the annotation found in Molecular Signatures Database (MSigDB) for tran-scription factors (TF).
